# Water without windows: Evaluating the performance of open cell transmission electron microscopy under saturated water vapor conditions, and assessing its potential for microscopy of hydrated biological specimens

**DOI:** 10.1371/journal.pone.0186899

**Published:** 2017-11-03

**Authors:** Cathal Cassidy, Masao Yamashita, Martin Cheung, Chola Kalale, Hidehito Adaniya, Ryusuke Kuwahara, Tsumoru Shintake

**Affiliations:** Quantum Wave Microscopy, OIST Graduate University, 1919-1 Tancha, Okinawa 904-0495, Japan; Institute of Materials Science, GERMANY

## Abstract

We have performed open cell transmission electron microscopy experiments through pure water vapor in the saturation pressure regime (>0.6 kPa), in a modern microscope capable of sub-Å resolution. We have systematically studied achievable pressure levels, stability and gas purity, effective thickness of the water vapor column and associated electron scattering processes, and the effect of gas pressure on electron optical resolution and image contrast. For example, for 1.3 kPa pure water vapor and 300kV electrons, we report pressure stability of ± 20 Pa over tens of minutes, effective thickness of 0.57 inelastic mean free paths, lattice resolution of 0.14 nm on a reference Au specimen, and no significant degradation in contrast or stability of a biological specimen (M13 virus, with 6 nm body diameter). We have also done some brief experiments to confirm feasibility of loading specimens into an *in situ* water vapor ambient without exposure to intermediate desiccating conditions. Finally, we have also checked if water experiments had any discernible impact on the microscope performance, and report pertinent vacuum and electron optical data, for reference purposes.

## Introduction

Atomic resolution transmission electron microscopy (TEM) of specimens in gas ambients, without enclosing windows, is well established in materials science, for a variety of gases and pressure values [1, 2]. However, the specific case of water vapor, with pressures in the saturation regime, and its effect on microscope performance, has not been studied systematically to date. Saturated water vapor is a very particular case of immense importance, in that it allows liquid water, and/or hydrated biological specimens, to be maintained in an equilibrium state. Slight increases or decreases in pressure cause controlled condensation or evaporation, respectively. In this saturation pressure regime, utilization of high resolution open cell gas microscopy, so successfully demonstrated in materials science, could be a transformative approach for life science microscopy, allowing dynamic, *in situ* studies of specimens in controlled native-state conditions.

To evaluate the feasibility of working under saturated conditions, without enclosing windows, in a high resolution TEM, there are a number of initial questions which we attempt to answer directly with this work. Can water vapor, in the saturation pressure regime, be supplied, measured and reliably controlled in the specimen area of a modern, aberration-corrected TEM? If so, is the optical performance through the necessary water vapor pressures good enough to justify its use? Is the specimen stable under simultaneous water vapor and electron beam exposure? How does imaging performed through water vapor measure up against established cryo- or closed cell approaches? Does the microscope hardware suffer any short-term or long-term degradation in performance as a result of prolonged water exposure? In this first phase, we concentrate on the systematic study of water vapor in the specimen area of the TEM, and also perform preliminary trials on specimen loading schemes that avoid exposure to intermediate high-vacuum conditions. We leave the systematic study of liquid water and specimen hydration for the future.

### Background and theory

The conversion of water between the liquid and gas phases is a ubiquitous phenomenon, and it is important at the outset to distinguish between the two rather different regimes of boiling and evaporation. Boiling occurs when the vapor pressure of the liquid exceeds that of the *total surrounding pressure*, and the liquid water begins to vaporize throughout the three-dimensional bulk of the liquid (bubbling). Evaporation, on the other hand, is quite different. It is purely a surface phenomenon, which depends on the net rate of water molecules leaving and striking the liquid surface. As a result, it is completely independent of the other gas species which may be present in the surrounding ambient, and depends only on the *water vapor partial pressure*. This is central to our proposed electron microscopy experiments. If we strip out most of the other ambient gas components (which don’t help with controlling evaporation, but would contribute directly to electron beam scattering), and preserve only water vapor, we can achieve saturation conditions (0.6-2 kPa, depending on the temperature) at a fraction of standard atmospheric pressure (101 kPa).

Mathematically, the factors controlling evaporation have been expressed concisely by Cameron and Donald [3]. The numbers of H_2_O molecules entering (*strike rate*) and leaving (*escape rate*) the surface per unit area, are primarily defined by three variable factors: the temperature of the liquid water (T_*l*_), the temperature of the ambient water vapor (T_*v*_) and the number density of water molecules in the ambient vapor (n_*v*_):

$$\begin{array}{c} \text{strike}\;\text{rate}=n_{v}{\left(\frac{m}{2\pi kT_{v}}\right)}^{\frac{1}{2}}\int_{0}^{\infty }e^{\left(\frac{-mu^{2}}{2kT_{v}}\right)}udu=n_{v}{\left(\frac{kT_{v}}{2\pi m}\right)}^{\frac{1}{2}} \end{array}$$ (1)

$$\begin{array}{c} \text{escape}\;\text{rate}=n_{l}{\left(\frac{m}{2\pi kT_{l}}\right)}^{\frac{1}{2}}\int_{\epsilon }^{\infty }e^{\left(\frac{-mu^{2}}{2kT_{l}}\right)}udu=n_{l}{\left(\frac{kT_{l}}{2\pi m}\right)}^{\frac{1}{2}}e^{\frac{-\epsilon }{kT_{l}}} \end{array}$$ (2)

All variables are explicitly defined in the Supporting Information (S1 Appendix). Net evaporation can then be derived from the difference between the strike and escape rates, and with some manipulation, leads to an expression for the rate of liquid water mass loss per unit area, in terms of the water vapor partial pressure, *P*_*w*_:

$$\begin{array}{c} \frac{{\text{dm}}_{\text{E}}}{\text{dt}}={\left(\frac{kT_{l}}{2\pi m}\right)}^{\frac{1}{2}}\rho_{w}e^{\left(\frac{-\epsilon }{kT_{l}}\right)\left[1-{\left(\frac{T_{v}}{T_{l}}\right)}^{\frac{1}{2}}\frac{P_{w}}{X\left(T_{l}\right)}\right]} \end{array}$$ (3)

**Fig 1:**
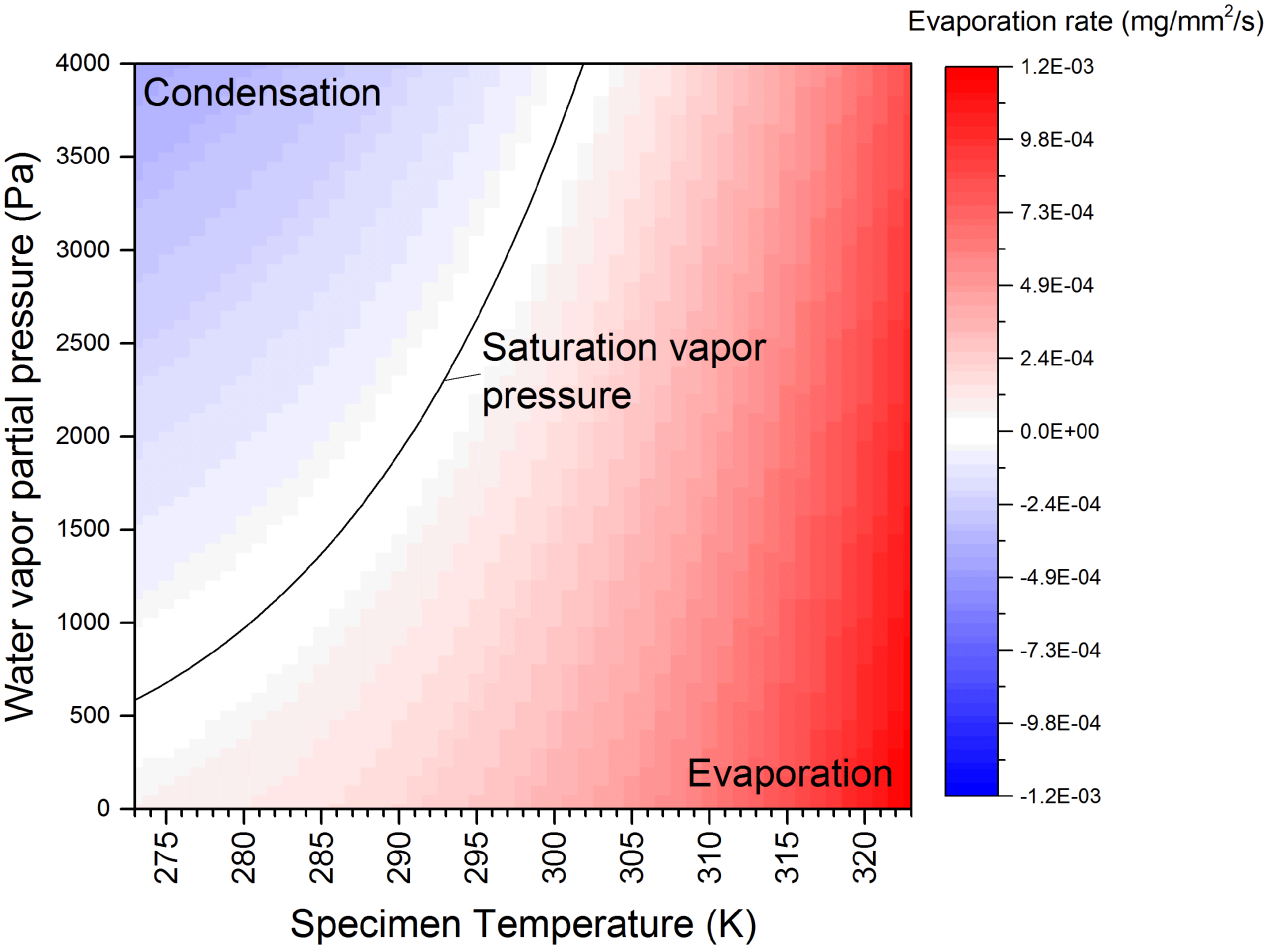
Net evaporation as a function of temperature and pressure, plotted using Eq 3. Evaporation rate per unit time and surface area, as a function of ambient water vapor partial pressure and specimen temperture (gas ambient temperature set to 298 K). The microscope used for this work allowed an ambient pressure of up to 2 kPa, indicating that the zero net evaporation condition is within range (with appropriate specimen cooling).

The relationship in Eq 3 is illustrated graphically in Fig 1 (in this case, for a fixed gas temperature of 298 K). The water vapor pressure at which evaporation and condensation are perfectly balanced is known as the saturation vapor pressure (SVP), and is indicated by the line centered in the white band in Fig 1. It is clear that the water vapor partial pressure required to achieve saturation depends very strongly upon specimen temperature, with lower liquid water temperatures corresponding to lower values of saturation vapor pressure. The gas temperature also influences SVP, causing SVP *decreases* as a function of gas temperature *increases*; however, the effect is considerably less pronounced than the liquid temperature case.

While the behavior of nanoscale specimens will presumably deviate from those of the bulk, nevertheless the bulk equations give us a basic guideline. We report a few relevant conditions (all assuming water vapor temperature of 298 K). With specimen temperature of 273 K, the saturation vapor pressure is 585 Pa (this is, in fact, the triple point of water). This condition is experimentally not very useful for microscopy purposes, but serves to identify the minimum water vapor pressure required to achieve saturation. More practically, with a specimen temperature of 277 K (i.e. 4°C, a common refrigeration temperature), the saturation vapor pressure is 729 Pa. This is a temperature range which should be experimentally practical with a suitable cooling holder. Regarding the upper pressure limit, the utilized microscope hardware imposed an upper gas pressure limit of 2 kPa, which would correspond to saturation vapor conditions with specimen temperature of 291 K. Changing the gas temperature will have a modest effect on the values reported above, but overall it is the specimen temperature that primarily governs the necessary water vapor partial pressure necessary to achieve saturation.

### Literature review

A wide variety of studies have already been published on liquids and gases in electron microscopy, so it is worthwhile to briefly summarize relevant previous work, and to highlight distinguishing features of the current study.

Water vapor exposure in Scanning Electron Microscopy (SEM) has been performed for many years [3], and several excellent reviews have already been published [3, 4]. While the experimental difficulties and achievable results are somewhat different in the TEM case, the physics of the water liquid/gas phase change are the same, as are the issues around loading specimens without exposure to conditions which would cause desiccation of the specimen [3]. For example, the recent review by Ross et al. briefly mentions using 30kV E-S(T)EM to image hydrated biological specimens under saturated water vapor conditions, with resolution on the order of 2nm [4]. Our underlying motivation is to explore possibilities for this configuration in high-end TEM, with all the associated capabilities for advanced imaging and spectroscopy afforded by this technique.

Closed liquid cell microscopy, which uses electron transparent membranes to separate the specimen from the adjacent vacuum, has made huge advances in recent years [5]. Two primary approaches have been followed. In the first case, patterned MEMS chips with defined thicknesses and geometries are utilized, and are available from a number of commercial manufacturers. Advantages include well-defined structure and possibilities for extended capabilities like flow, heating, or electrochemistry [5]. Disadvantages include the necessity of relatively thick enclosing windows and liquid layer, limited field of view (lateral and tilt), thickness variation owing to ill-defined bowing of membranes, and risk of rupture into a delicate microscope environment. In the second case, several research groups have successfully fabricated graphene liquid cells [6, 7]. This is hugely advantageous in terms of the “thin-ness” of both the water layer and the monolayer-thick graphene windows, but on the other hand is a rather tricky sample preparation process relying somewhat on fortune to produce adequate specimens.

Open cell transmission electron microscopy, involving introduction of gases into the specimen area of a differentially-pumped microscope, has also seen huge progress in recent years, particularly in materials science (see recent reviews [8, 9]). Several groups have performed quantitative experimental studies on the effect of selected gases, such as H_2_, N_2_ and O_2_, upon HRTEM [2, 10, 11] and EELS [12] measurements. These studies serve as quite comprehensive references, and include detailed reports on the effect of gas pressure levels, gas composition, microscope acceleration voltage, electron dose and total electron beam current, on the achievable microscope performance.

While quantitative studies, like those mentioned in the preceding paragraph, have not been performed to date for water vapor, nevertheless, some open-cell experiments utilizing water vapor have been performed. These range from the seminal 1974 work of Parsons, which demonstrated saturation vapor pressure imaging of wet specimens [13]. This work was performed on an older generation microscope, with micrograph fields of view on the order of tens of microns. Detailed optical performance values were not reported, but resolution on the order of 5 nm was mentioned. More recently, water vapor has also been introduced into modern, atomic-resolution capable microscopes, but in the sub-saturation pressure regime, and often in an “offline” mode (with the electron beam column valves closed while the water vapor was present). Yoshida et al. studied Pt catalysts in the presence of water vapor in the mPa range [14]. Miller acquired EELS data from 133 Pa of water vapor generated from various purities of source water, ranging from high resistivity Nanopore water to raw Mediterranean seawater [15]. Two studies from the Crozier group involved simultaneous exposure to elevated temperatures, optical light, and water vapor in the 67-133 Pa range, with durations up to 60 hours [16, 17]. The highest water vapor pressure that we have noted in a modern, open cell microscope has been 500 Pa, for up to 3 hours (with the electron beam blanked during water vapor exposure) [18]. Referring back to Fig 1, none of these existing studies with modern high resolution microscopes have investigated the saturation regime (>0.6 kPa).

Against this background of previous work, distinguishing features of the current work are that we have performed “beam on” studies through water vapor in the saturation pressure regime (>0.6 kPa), in a modern microscope capable of sub-Å resolution. We have systematically studied achievable pressure levels and stability, gas purity, and elimination of carrier gases, effective thickness of the water vapor column and associated electron scattering processes, and the effect of gas pressure on electron optical resolution and image contrast. We have also done some brief exploration of transfer schemes to load specimens into an *in situ* water vapor ambient without exposure to intermediate desiccating conditions. Finally, we have also checked if water experiments had any discernible impact on the microscope performance, and report pertinent vacuum and electron optical data, for reference purposes.

## Materials and methods

**Fig 2:**
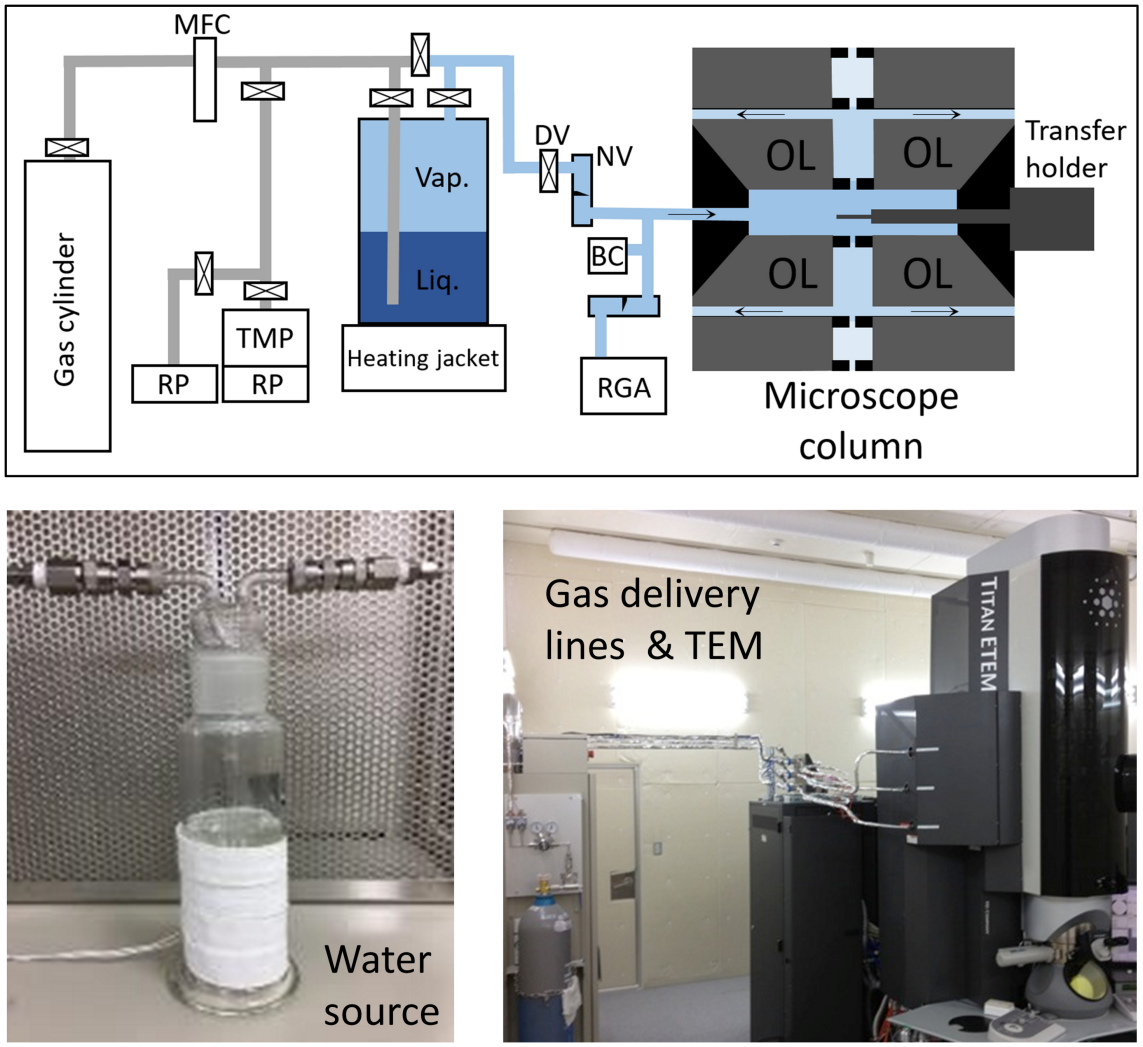
Schematic diagram (top) and photographs (bottom) of the experimental set-up. The acronyms in the schematic diagram refer to: TMP (Turbo Molecular Pump), RP (Roughing pump), RGA (Residual Gas Analyser), BC (Barocel pressure gauge), DV (Diaphragm valve), NV (Needle valve), OL (Objective lens).

The experimental set-up is illustrated in Fig 2. Experiments were executed using an environmental TEM (Titan G2 80-300, Thermo Fisher Scientific (formerly FEI Company)), operated at 300 kV [10]. This microscope was selected based upon its ability to supply gases to the specimen area, but its hardware was configured for materials science, rather than life science, specimens. As such, the microscope was not equipped with typical life science components such as a high sensitivity camera, automated low-dose exposure control, nor a contrast-enhancing phase plate. In detail, the microscope was equipped with a Schottky field emission gun (XFEG), differentially pumped specimen area, post-specimen image C_s_-corrector (Cetcor, CEOS Gmbh), post-column electron energy loss spectrometer (Quantum 966, Gatan), and CCD camera (UltrascanXP, 2k x 2k pixels, Gatan). Gas pressure was measured using a capacitance manometer (Barocel^®^ Model 622, Edwards Limited), and gas composition was measured using a residual gas analyzer (PrismaPlus^™^ QME-220, Pfeiffer Vacuum). A plasma cleaner (Evactron^®^ 45 De-contaminator, XEI Scientific, Inc.) was available to apply specimen area cleaning after execution of experiments.

Specimens were loaded using standard side entry specimen holders (FEI low-background holder, and Gatan 648 transfer holder). A standard calibration sample (S106 cross-grating, Agar), consisting of polycrystalline Au islands on an amorphous carbon support, was utilized for optical resolution evaluation during water vapor exposure. M13 virions, with 6 nm diameter, were utilized for evaluating the biological specimen stability and contrast in a water vapor ambient. A holder with transfer and cooling capability was not available for this work, so all experiments were executed at room temperature (for calculation purposes, assumed to be 298 K).

Water vapor was supplied from an external bath, as shown in Fig 2, containing ultra-pure water (UPW) with resistivity of >18MΩ.cm. This configuration is based upon that reported by Yoshida et al. [14]. The water dewar ambient and supply line were pre-evacuated using an independent pumping line. Reduction of ambient gas pressure above the liquid water was sufficient to cause evaporation and create adequate supply of water vapor [15]. It was possible to heat the supply line to regulate the vapor temperature (T_*v*_), as introduced in Eq 1, as well as to heat the water bath to increase the supply of water vapor, if necessary. It was also possible to flow carrier gases through the water vapor bath if desired (not used in these experiments).

Full information on settings for EELS acquisitions, HRTEM resolution tests, and biological specimen preparation and image acquisition are included in the Supporting Information (S2 Appendix).

## Results

### Gas delivery and measurement

As noted in the introduction, it is not the total pressure, but rather the partial pressure of water vapor, independently, that governs net surface evaporation or condensation rates [3]. This means that knowledge of the total system pressure is not sufficient—the specific partial pressure of water vapor must be known and controlled. With this in mind, we have focused our investigation on pure water vapor, by eliminating residual atmospheric gases from the supply path, and without using carrier gases in the supply methodology. The experimental set-up is designed to allow easy addition of defined flows of inert carrier gases to the water vapor line at any time, should a particular gas mixture be desired later (for example, a buffer of an inert, weakly scattering species like He, could be included to create a controlled offset between the saturation vapor pressure, and the boiling pressure).

**Fig 3:**
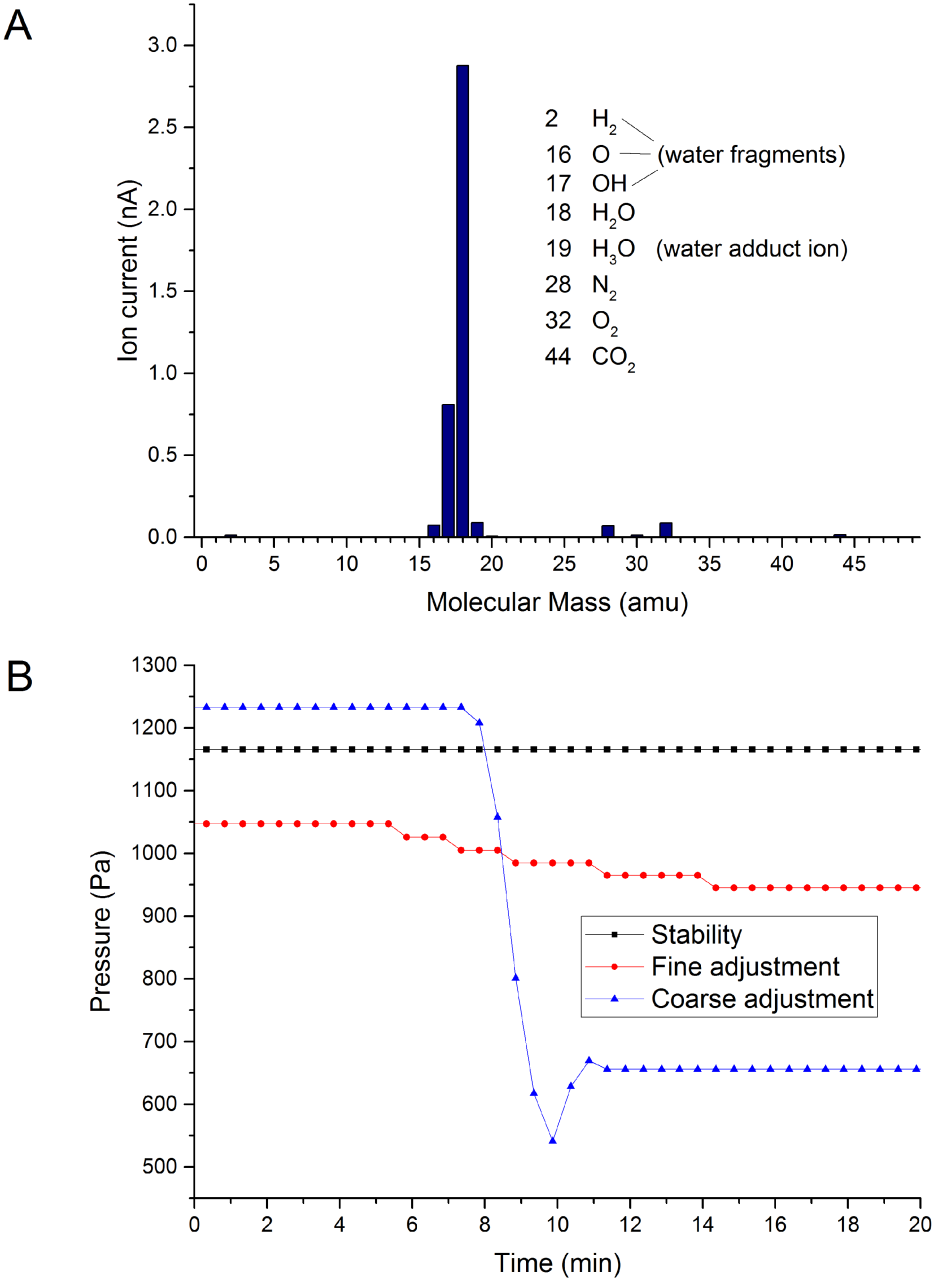
Gas composition and pressure control. A. RGA spectrum acquired at a specimen area pressure of 1.98 kPa (room temperature). Most of the detected ion current is associated with water vapor (at molecular mass 18) and its ionization fragments. Small peaks from residual nitrogen, oxygen and carbon dioxide are also detected. B. Curves demonstrating the system control over the gas pressure. We show the optimum stability of the system when the set-up parameters are fixed (no fluctuations are detected, within the measurement sensitivity of the gauge, over periods exceeding twenty minutes). We also show the response under fine adjustment of the inlet needle valve, demonstrating fine control and identifying that the smallest measurable pressure shift is ∼20 Pa. We also demonstrate coarse adjustment, to a defined setpoint over a wide pressure differential, demonstrating the coarse control, transition time and stability after adjustment.

The gas delivery set-up was as shown previously in Fig 2. The composition of the supplied gas was examined using the *in situ* Residual Gas Analyzer (RGA). Immediately after replenishing the water supply, significant levels of residual atmospheric gases (*O*_2_, *N*_2_, CO_2_) could be detected (S1 Fig). However, these could be removed by pumping the system for a few hours, and this process did not need to be repeated again until the next atmospheric exposure for water bath replenishment. Once the set-up was optimized and the dewar and supply line had been fully evacuated, water vapor and associated fragments were the only significant species detected with the RGA. This can be seen in Fig 3A. To confirm the correct functioning of the RGA, we also acquired reference spectra from high (6N) purity nitrogen, oxygen and hydrogen (S2 Fig). We also acquired RGA spectra as a function of varying gas pressure in the range 0 kPa to 2 kPa, for nitrogen, oxygen, and hydrogen (S3 Fig). Note that, in this dynamic case, the relationships between indicated pressures and RGA ionization currents were not linear (as explained in the Supporting Information). We will return to this topic in the next section.

Having verified the gas composition, the next step involved evaluating control of pressure setpoint and stability of the system. This is important—as illustrated graphically in Fig 1, to image hydrated specimens we must carefully maintain the specimen in the “white zone” at or close to the saturated vapor pressure condition. The approach that we used was to achieve a high pressure state by regulation of supply bath valves and temperature, we then utilized the microscope gas inlet needle valve to step down the pressure to the desired setpoint in the specimen area. Examples of pressure control are shown in Fig 3B. We firstly show the best achievable system stability (black squares). In this example, the system remains stable for over twenty minutes at 1165 Pa (within the measurement sensitivity of the gauge), and such stability persisted over longer time scales. Next, to demonstrate fine control, the inlet needle valve was minutely adjusted to change the pressure in a controlled fashion (red circles), and which clearly shows that the smallest measurable interval is ∼20 Pa. Presumably, the actual control achievable with the needle valve is better than this. Finally, we show coarse adjustment of the supplied water vapor pressure, demonstrating the transition time and stability after adjustment (blue triangles). Although not utilized for these experiments, the system control software provided an automated function to lock the pressure to a user-defined setpoint, via a feedback loop between the pressure gauge and with the motorized inlet needle valve. These combined results suggest that the water vapor supply set-up provides sufficient control and stability to achieve and maintain saturation pressure conditions.

### EELS measurements from water vapor

While the results in the previous section are encouraging, they are system-level measurements performed with gauges (pressure and mass spectrometry) that are physically displaced from the electron beam-specimen interaction area, and in general gas fluid dynamics are notoriously complex. As noted in the preceding section, we observed some time lag and non-linearity between the responses of the *in situ* RGA and Barocel pressure gauge, to defined changes in gas composition. Given the requirement to tune the conditions carefully to the saturation vapor pressure, it is important to have direct feedback on the gas composition and pressure, right from the location of interaction between the specimen and electron beam.

**Fig 4:**
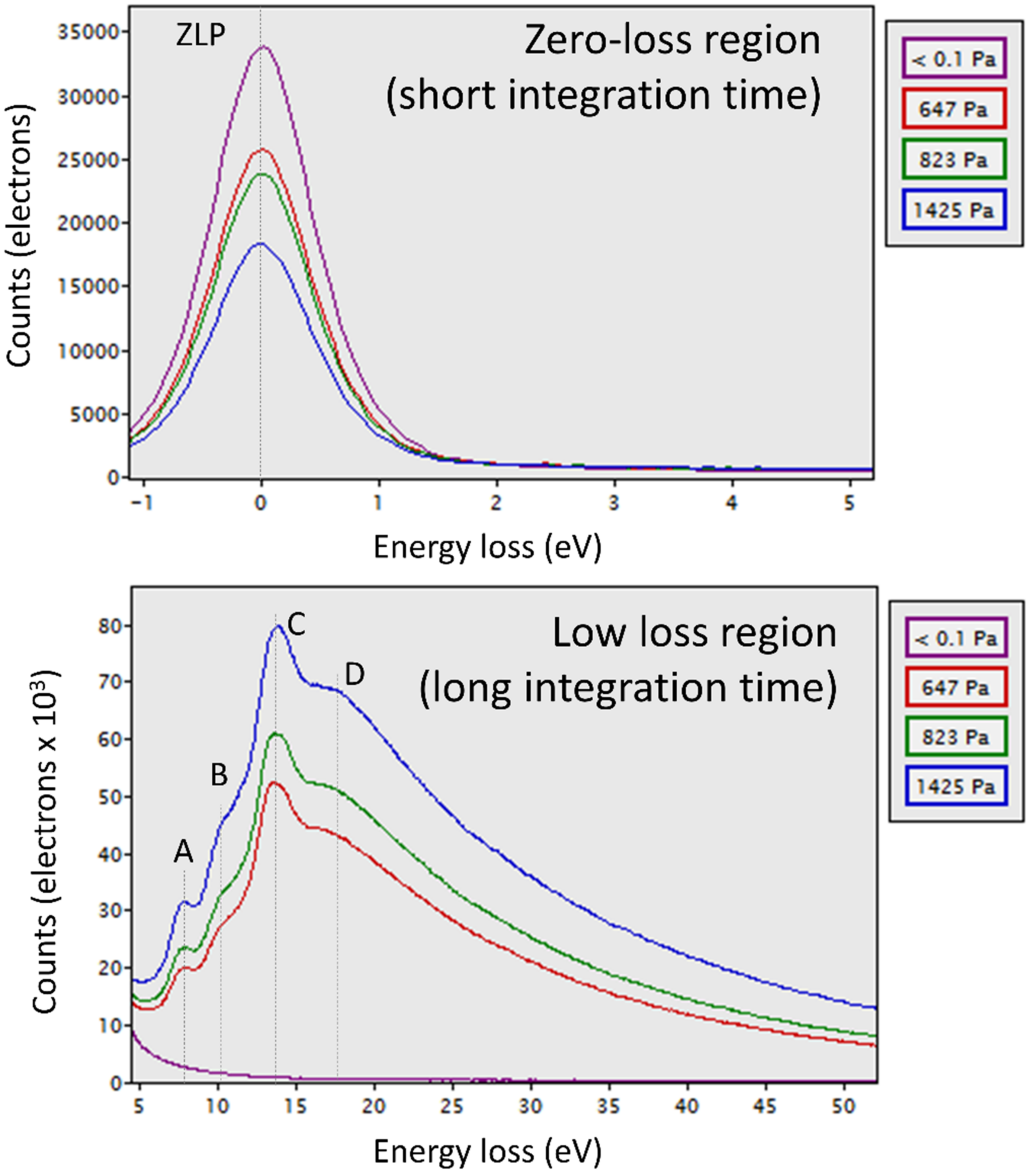
Zero-loss and low-loss EELS spectra of water vapor at various pressures (300kV, room temperature). The zero-loss-peak FWHM is approximately 1 eV, and the observed loss peaks are centered at 7.7 eV, 10.1 eV, 13.6 eV and 18.1 eV. These values are consistent with prior reports on ultraviolet absorption edges in water vapor (see main text). Zero-loss peak intensity decreases, and energy loss intensity increases, as a function of water vapor pressure, as expected. The integration times were 1ms (zero-loss region) and 200ms (low-loss region), respectively.

Electron energy loss spectroscopy (EELS) was therefore employed to obtain information on the electron beam interaction with the water vapor, directly from the specimen region. Illustrative examples of EELS acquisitions at selected nominal pressures, are introduced in Fig 4. The zero-loss and low-loss electron energy loss spectra were acquired “simultaneously”, but with different integration times, using the established dual EELS function [19]). The zero-loss peak full-width half-maximum, with the utilized settings, was about 1 eV, and gives an indication of the resolution of the acquired spectra. As expected, the intensity of the zero-loss peak decreases, and the low-loss peaks increases, as the gas pressure increases. Obviously, this intensity distribution gives important information on the scattering properties of the gas, and will be discussed in detail in the next paragraph. Considering firstly the appearance, the general form of the low-loss spectrum is consistent with previous reports on absorption spectra of water [15, 20]. Four distinct peaks can be distinguished (marked A-D in Fig 4). The expected electronic transitions, from water molecules in vapor form, have previously been documented ([21] and references therein) as: 7.4 eV (excitation from 1b_1_ molecular orbital to 3sa_1_ Rydberg state), 9.7 eV (3a_1_ to 3sa_1_), 13.4 eV (1b_2_ to 3sa_1_, and 3a_1_ to 4sa_1_ and nd states) and 17.5 eV (1b_2_ to 4sa_1_). Our experimental EELS values agree quite well with these values. Although beyond the scope of this work, a methodology for quantitative gas compositional analysis using low-loss EELS spectra has already been published, with results obtained for other gases [12]. This water vapor EELS data could be utilized in a similar fashion to quantify the water vapor content in gas mixtures. With this in mind, we provide our raw spectral data from high purity water vapor (S1 Dataset, and corresponding acquisition conditions S2 Appendix), in case it is helpful for others for quantification of water content in gas mixtures. Furthermore, it should be clear that dedicated EELS experiments (for example, with higher energy resolution, different electron dose rates, and different gas conditions), in conjunction with quantitative simulations, such as reported by [22, 23], could yield both fundamental and practical insight into the state of the water molecules (such as ionization state, and vapor cluster nucleation state). We leave this for future work.

**Fig 5:**
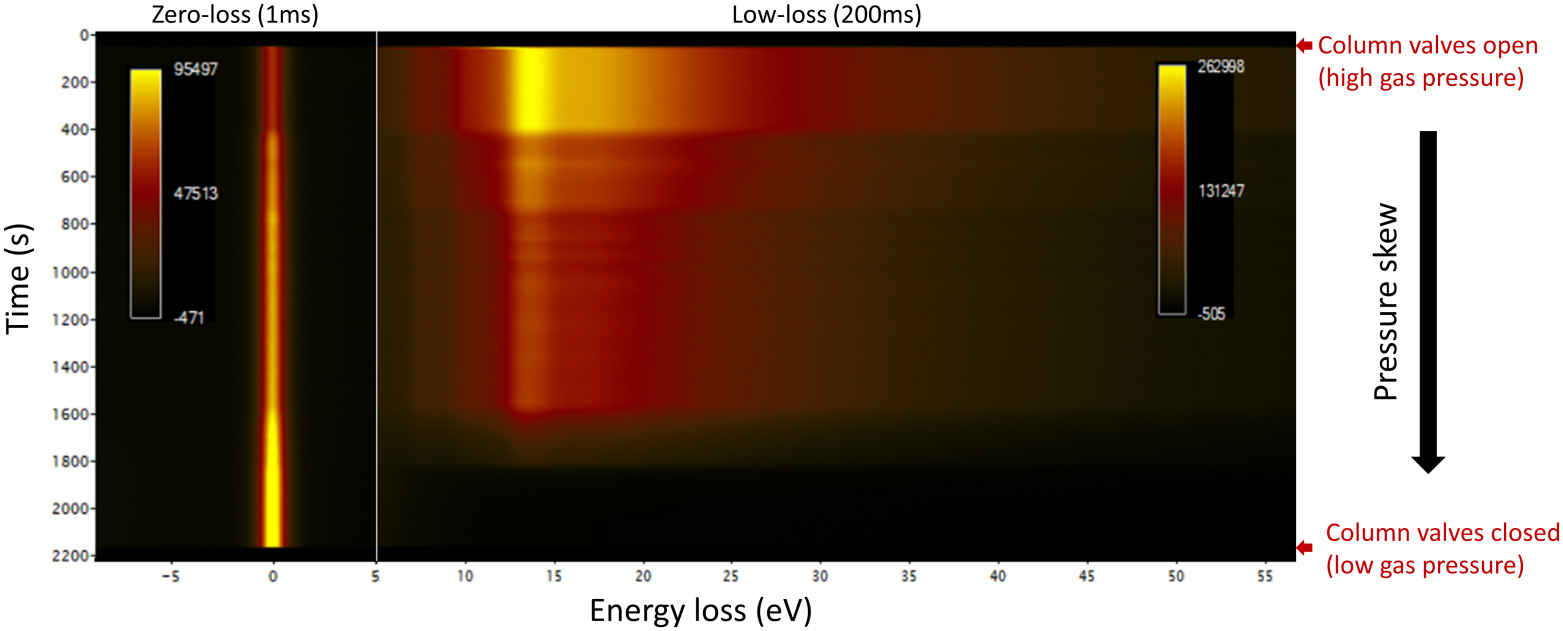
Time-resolved EELS spectra, as a function of decreasing water vapor pressure. Zero-loss and low-loss EELS spectra were acquired as a function of time, during a gas pressure skew, reducing to vacuum levels from an initial pressure of 1.4 kPa. The exact pressure skew is included in S4 Fig. The column valve open/close events allow the EELS spectra and pressure gauge readings to be precisely synchronized.

Of more direct relevance to the current work is that EELS data, coming right from the target specimen area, can be acquired dynamically and utilized to monitor actual conditions in the specimen area, as a function of water vapor supply conditions, into the rather complex differentially-pumped environment. This is shown in Fig 5, in the form of a spectrum image, processed and displayed using custom Digital Micrograph scripts [24, 25]. EELS spectra were acquired sequentially, and integrated together to form a 2D image with energy loss on the x-axis, time on the y-axis, and EELS counts given by the z value (pixel color). In this example, zero-loss (1 ms) and low-loss (200 ms) spectra were acquired, and to avoid data overflow, every 10th frame was stored (resulting in an interval time of 4.3 seconds per EEL spectrum, defining the y-axis time step). Energy spectra shown in Fig 4 are equivalent to horizontal (*energy*) slices through Fig 5, but it should be clear that we can also acquire vertical slices, which map the evolution of defined energy windows as a function of *time*. In the first instance, we can confirm the general agreement and synchronicity of the signals from the system-level Barocel gauge and the electron scattering from the specimen area gas column, as shown in S4 Fig. Within our measurement resolution, signals are well-synchronized and correlated, in agreement with previous computational fluid dynamics simulations [26]. The distinct measurement points are within a uniform, unobstructed pressure chamber in this case [26], and the previously noted poor correlation between specimen area pressure changes and RGA readings, was not an issue in this case. Thus, having validated the synchronicity between the pressure gauge and EELS readings, we can proceed with more quantitative analysis of electron scattering as a function of nominal pressure.

**Fig 6:**
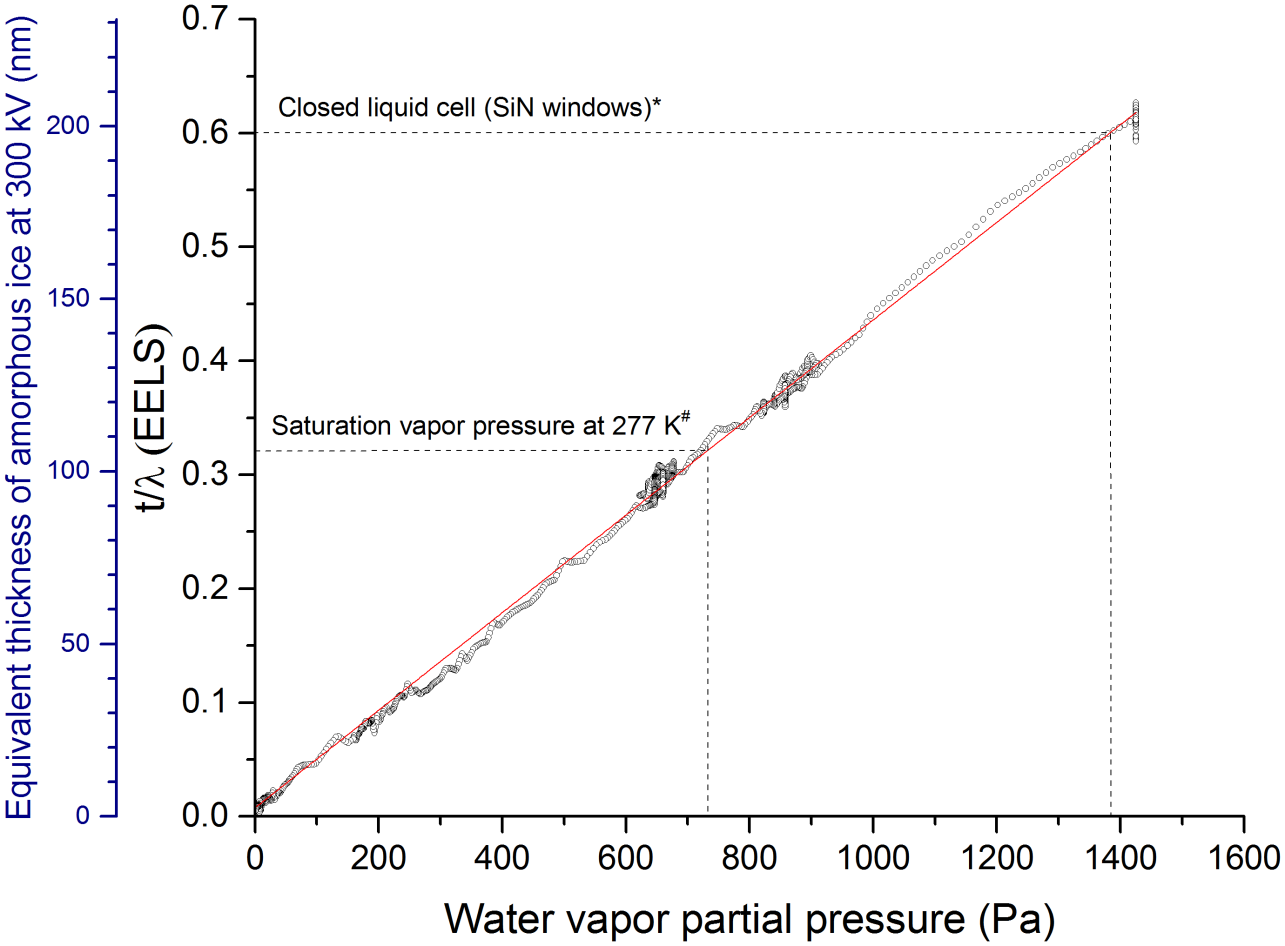
Effective thickness of water vapor for 300 kV electrons, expressed in terms of t/λ, as a function of indicated pressure. Effective thickness has been calculated according to ${\left(\frac{\text{t}}{\lambda }\right)}_{\text{P}}=ln\frac{\int I_{Vac}dE}{\int I_{ZLP(P)}dE}$, with full details in S3 Appendix. The effective thickness is quite linear as a function of pressure. For comparison purposes, we have also included the equivalent thickness of amorphous ice (assuming an inelastic mean free path of 330 nm at 300 kV [28]. We indicate (*) a literature value for the effective thickness of enclosing SiN membranes in a conventional closed liquid cell, from [27]. We have also marked (^#^) the effective water vapor thickness corresponding to saturation vapor pressure at 277 K (0.32 at 729 Pa).

Evaluation of electron scattering from the water vapor column (defined by the electron beam propagation through the gas ambient) is a rather complex problem. Therefore, we have explored different experimental beam optics configurations and data analysis methodologies (all of which are included in S3 Appendix). The summary result, following this evaluation, is shown in Fig 6 (some reference values from the literature have also been overlaid, for comparison [27, 28]). In this case, we have employed a very well-characterized collection angle geometry for data acquisition [29], and calculated the effective thickness as a function of pressure using:

$$\begin{array}{c} {\left(\frac{\text{t}}{\lambda }\right)}_{\text{P}}=ln\frac{\int I_{Vac}dE}{\int I_{ZLP\left(P\right)}dE} \end{array}$$ (4)

All variables are explicitly defined in S3 Appendix. First, it is clear from Fig 6 that the effective thickness is quite linear with pressure, indicating a straightforward relationship between macroscopic pressure measurement and electron scattering by the gas. Second, these effective thickness values allow us to deduce, expressed as an areal density, the number of water molecules intercepted by the electron beam as a function of pressure (S4 Appendix), and with some assumptions, to confirm that the actual pressure in the specimen area, as encountered by the electron beam, is consistent with the reading of the system-level pressure gauge (S4 Appendix). Finally, and most importantly, the effective thickness of a column of water vapor to 300 kV electrons, as a function of pressure in the saturation regime, is documented for the first time, and is competitive with existing ice-embedded or closed cell approaches. For example, for a pressure value of 729 Pa, corresponding to zero net evaporation at temperatures of 277 K (liquid) and 298 K (gas)(Fig 1), the measured effective thickness is 0.32 (Fig 6). These gas t/λ values, in the saturation regime, compare favorably with reported values for solid state windows (e.g. t/λ ∼ 0 (monolayer graphene sandwich) to t/λ ∼ 0.6 [27]) and 100 nm thick amorphous ice (t/λ ∼ 0.3 [28]). This suggests that open cell water vapor can exhibit comparable scattering performance to these established methods. In comparing with established methods, the role of the effective thickness calculation methodology, and the characteristics of image formation and contrast in a vapor ambient, are important considerations and are discussed further in the Discussion section.

### Microscope resolution as a function of water vapor pressure

**Fig 7:**
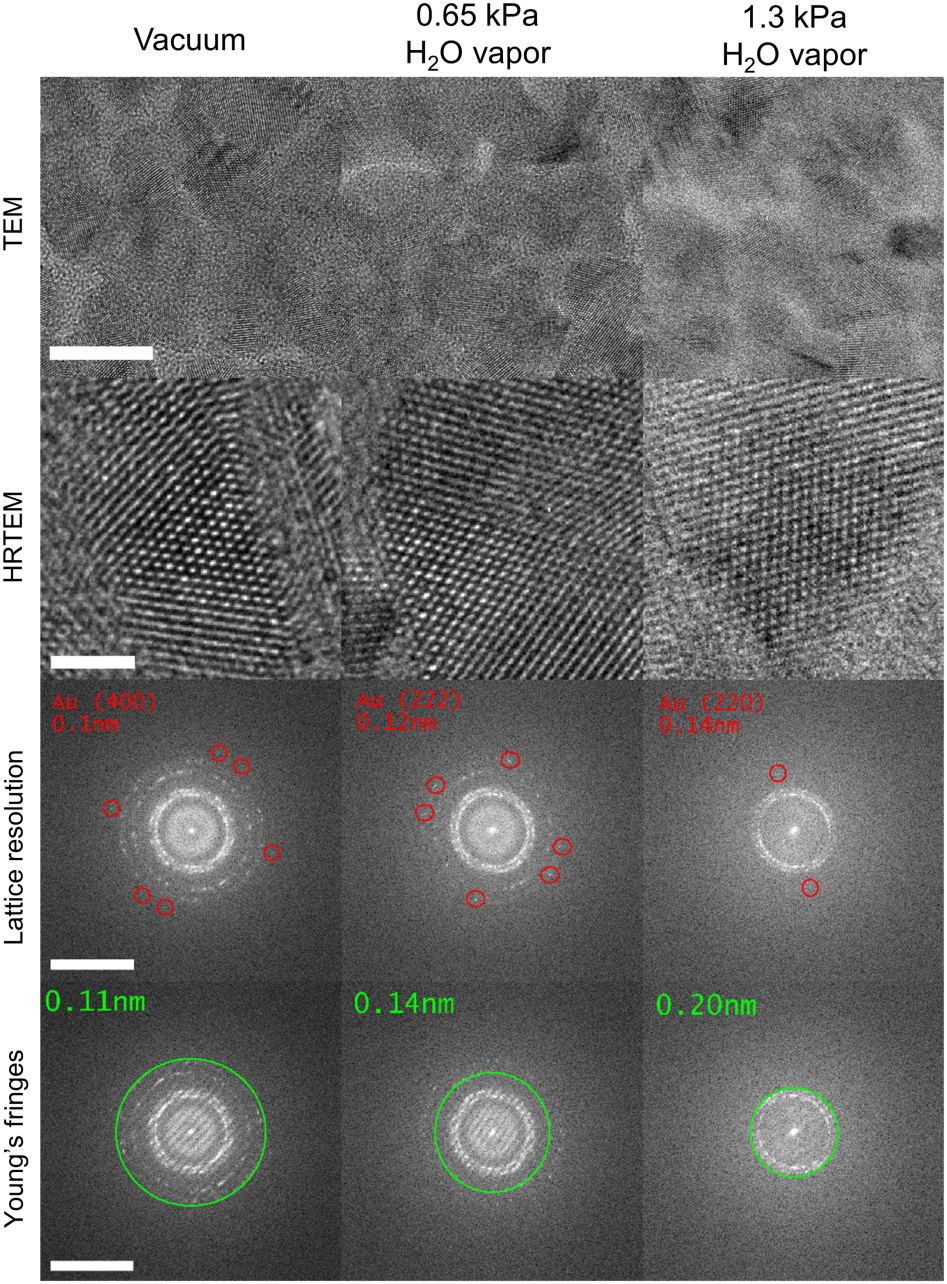
Microscope resolution as a function of water vapor pressure in the specimen area. Scale bars are 10 nm, 2 nm, 10 nm^−1^ and 10 nm^−1^, respectively. The specimen is polycrystalline gold on a carbon support. We observed maximum lattice resolution of 0.1 nm from Au(400), 0.12 nm from Au(222), and 0.14 nm from Au(220), and Young’s fringes resolution of 0.11 nm, 0.14 nm and 0.2 nm, at water vapor pressures of <1 mPa (vacuum), 0.65 kPa and 1.3 kPa, respectively. Atomic resolution is readily attained over this water vapor pressure range.

Having demonstrated that water vapor can be supplied in a controlled fashion, and having gained some insight into electron scattering effects of the water vapor column, the next step was to evaluate the effect of the water vapor ambient on the imaging performance of the microscope. We began with a standard crystalline Au calibration sample to measure changes in the optical performance of the microscope as a function of water vapor pressure (we will consider biological specimens in the next section). Results are shown in Fig 7. There is a wide range of definitions and methods for measuring resolution, and we have reported both lattice fringe resolution [10] and Young’s fringes [11]. The microscope was first aligned in vacuum, including aberration correction, to confirm the specified resolution (<1Å at 300kV). HRTEM micrographs were then acquired at the indicated gas pressures, without any adjustment of the electron optics. It was observed that the measured electron beam current decreased as a function of gas pressure (as expected based on the TEM-mode EELS data included in S3 Appendix). In the first case, the qualitative image appearance was not dramatically changed as a function of gas exposure, and the Au lattice is readily discernible under all gas pressures. In Fourier space, we could resolve maximum spatial frequencies from lattice fringes of Au(400), Au (222), and Au(220), with spacings 0.1 nm, 0.12 nm and 0.14 nm, at water vapor pressures of <1 mPa, 0.65 kPa and 1.3 kPa, respectively. We also obtained Young’s fringes in the usual fashion [11], and observed contrast to spatial frequencies equivalent to resolutions of 0.11 nm, 0.14 nm and 0.20 nm, at pressures of <1 mPa, 0.65 kPa and 1.3 kPa water vapor, respectively. It is clear that the electron optical performance of the microscope remains suitable for a range of life or physical science applications, even in the presence of relatively high water vapor pressures in the saturation regime. For this particular specimen material set (Au islands on amorphous carbon), we encountered no noticeable problems with etching or decomposition of the sample, under the combined electron beam/water vapor exposure. This is a promising first sign for biological specimen microscopy, given that in these resolution tests, the support material was carbon, and much higher electron doses were applied than would usually be employed in life science electron microscopy.

Note that all prior works with inert gases [2, 10, 11] found that minimizing the electron dose directly improves achievable resolution, an effect that was consistently attributed to ionization and charging of the gas by the electron beam. While we have not studied this topic systematically, we have also confirmed that imaging performance improves with reduced electron beam current in water vapor (S5 Fig). This provides an indication that the highly sensitive microscopy technologies, which are well-established in the cryo-EM community, would bring significant advantages if applied also for *in situ* gas microscopy, even in materials science applications.

### Bio-specimen contrast

As noted in the introduction, imaging through water vapor in the saturation pressure regime could be a transformative strategy for microscopy of life science specimens, and is indeed the primary motivation which triggered this work. With this in mind, we did some trials with M13 filamentous phages as a test specimen. M13 is a common bacteriophage, that is, a virus which exclusively attacks bacteria, and is thus harmless to humans. It is, however, intensively researched for uses in drug delivery [30], cancer research [31], and nanotechnology [32]. It has a typical diameter and length of 6nm and 1000nm [31], respectively, and as such, serves as an appropriate test for image contrast and stability evaluation in water vapor. As already mentioned, we did not have available hardware to cool the specimen, so we do not make any claims that the specimen is still hydrated in this work. Furthermore, we did not use a microscope optimized for beam-sensitive specimens, so we do not consider these images competitive with state-of-the-art life-science microscopy. We should also mention that the reported dose rates were the output values from the manufacturer software, and were not independently calibrated or tightly controlled, so should be considered only as an indication of the approximate dose rate. Our goal at this stage was only to get a first indication the gas-embedded specimen contrast and stability, rather than to prove specimen hydration or quantify resolution or contrast enhancements.

**Fig 8:**
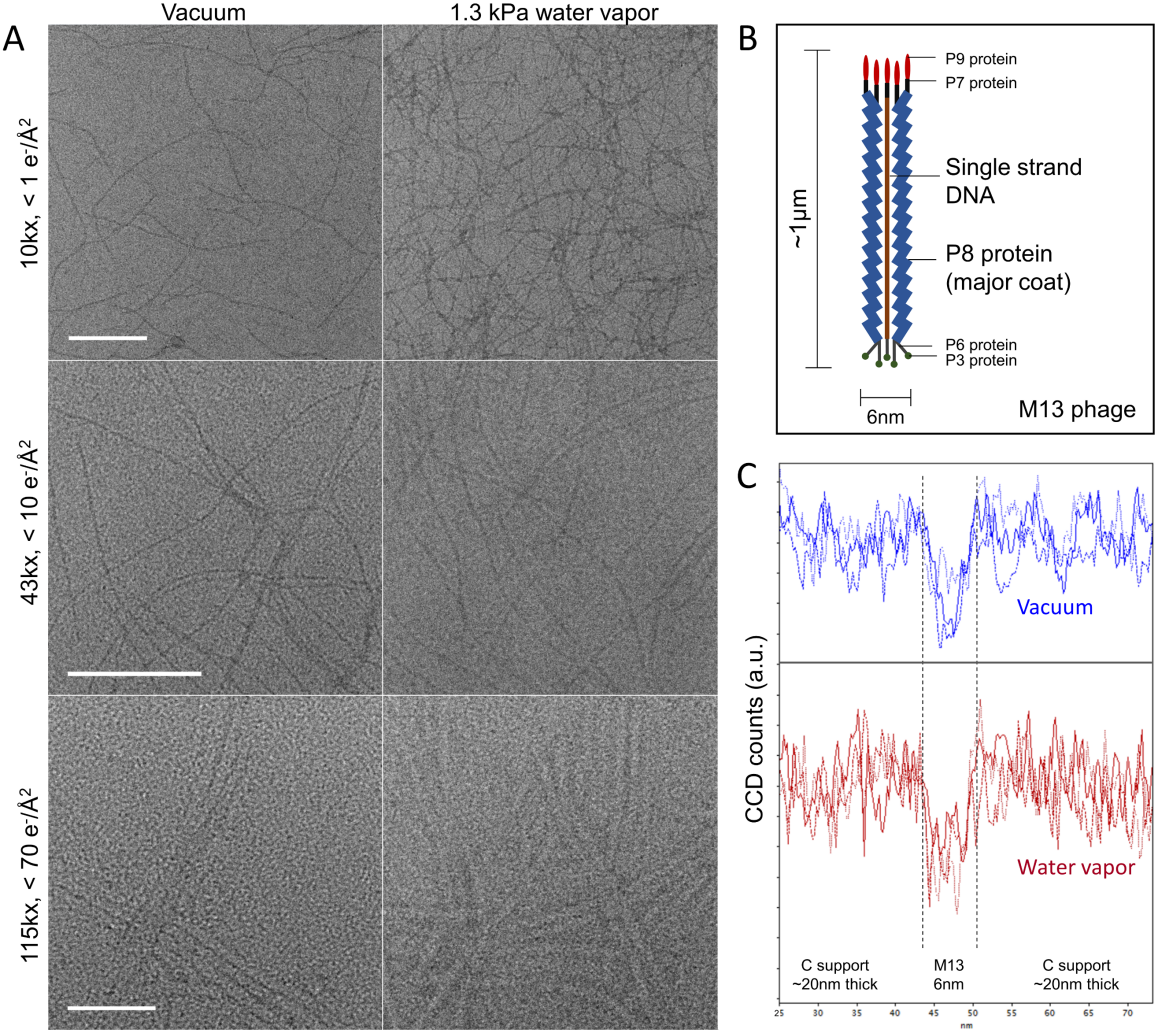
Biological specimen contrast when imaged through water vapor in the saturation pressure regime. A. M13 phage (unstained), imaged at 300 kV in high vacuum and in 1.3 kPa water vapor ambient, at magnifications of 10kx, 43kx and 115kx (Scale bars are 500 nm, 200 nm, 50 nm, respectively). B. Sketch of M13 phage structure, based upon that described in [31]. C. Line profiles extracted across several individual filamentous phages, for the vacuum and 1.3 kPa ambient cases. Individual profile locations and data are included in S2 Dataset.

Representative images are shown in Fig 8A, in vacuum and in a 1.3 kPa water vapor ambient, alongside a sketch of M13 structure [31] in Fig 8B. First, considering the images acquired in standard high vacuum conditions, the overall appearance is consistent with previous TEM images of M13 phage in the literature [33]. Second, considering the images acquired through water vapor, it is apparent that the individual M13 filaments are still visible, and with similar contrast to the vacuum case. Crucially, we did not encounter any problems with specimen stability under simultaneous electron beam and water exposure. With deliberately excessive exposure, we observed a speckle texture developing on the surface (S6 Fig). We include two videos in the Supporting Information (S1 and S2 Videos), with different microscope and specimen conditions, to give the reader a direct insight into the specimen appearance and stability as a function of time and continuous electron exposure, in a water vapor atmosphere.

We do not deem it appropriate to perform quantitative analysis or make definitive judgements based upon these images—this should be performed in a dedicated study with appropriate microscope settings and careful control over specimen preparation, defocus levels and cumulative electron dose. We nevertheless extract some line profiles across the body of the filamentous phage, in order to get a first indication of the signal-to-noise ratio in vacuum and in gas. Results are shown in Fig 8C. The profile extraction locations, and plots from each of the individual profiles, are included in S2 Dataset. Following [34], the specimen contrast can be fully and unambiguously evaluated by considering the ratio of the signal variance in the support area, and the difference of the mean values in object (virion) and background (support) regions. In short, it appears that specimen contrast is not severely compromised when imaged through water vapor in this pressure range. We will return to this topic in the Discussion section, with some comments regarding the theory of image formation in gas atmospheres.

### Specimen transfer trials

Aside from demonstrating the necessary water vapor ambient, another critical component of this work is to ensure that the specimen can be loaded from bench to microscope, without intermediate (high vacuum) periods which would cause rapid desiccation (as discussed in detail by [3]). While we did not have access to a cooling specimen holder which would be necessary to preserve liquid water in the available pressure range, nevertheless we performed some preliminary trials to explore the feasibility of loading a specimen from the bench into the already primed microscope water vapor ambient, without exposure to ultra-high vacuum at intermediate steps. For these trials, we used a transfer holder that allowed specimen mounting under atmospheric pressure, and sealing in an internal chamber. The (sealed) holder could then be loaded into the microscope, and once appropriate ambient conditions were confirmed, the internal chamber could be opened to release the atmospheric pressure and equilibrate with the ambient microscope pressure. This involved finding a sequence which worked within the specific constraints imposed by the microscope hardware and interlocks, as discussed in detail below.

**Fig 9:**
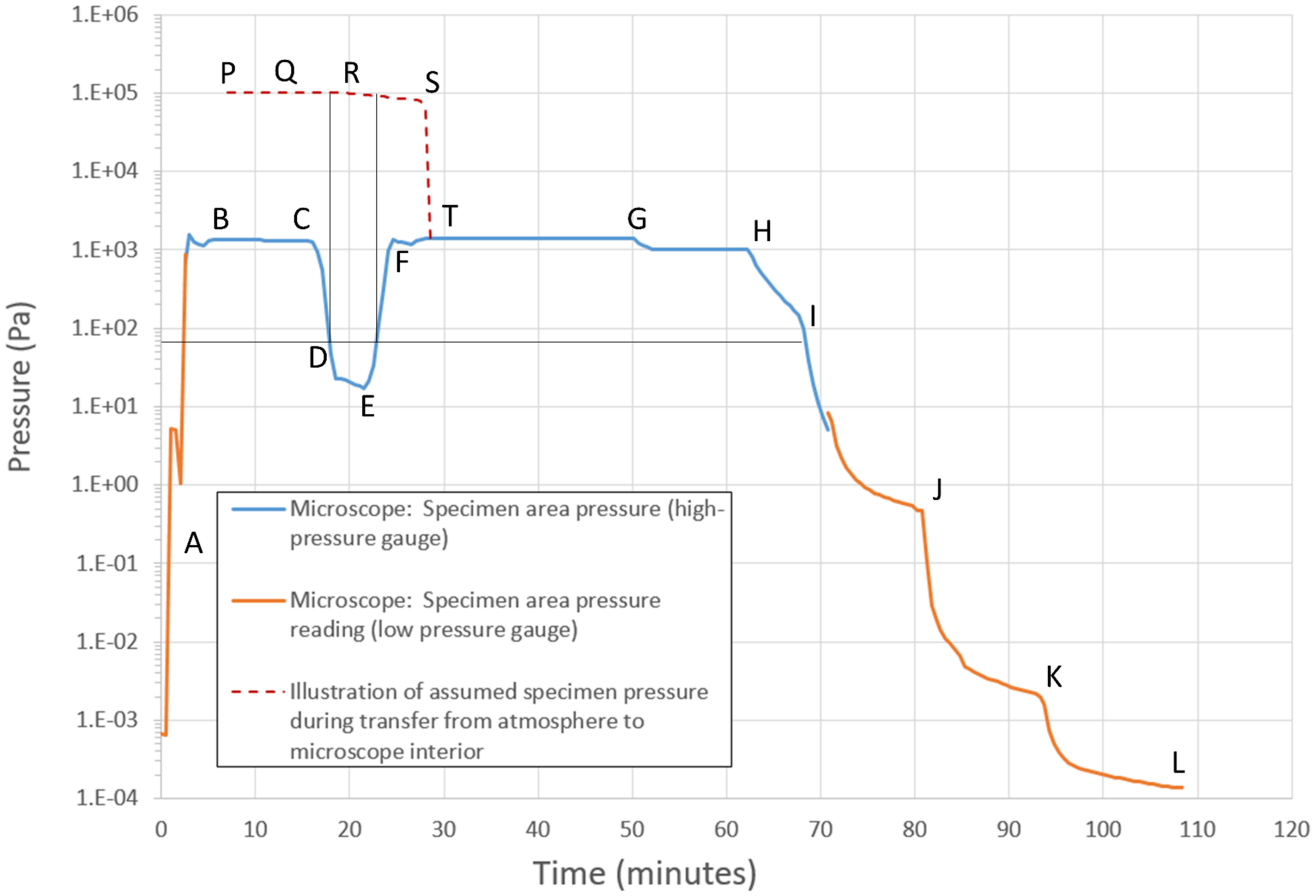
Demonstration of feasibility of specimen loading into a water vapor ambient in the microscope, without exposure to desiccating conditions. We confirmed a loading sequence that allowed the specimen to be loaded into the gas-laden microscope, without exposure to intermediate high vacuum conditions. Letters A-L and P-T are referred to in the text. It took approximately 5 minutes to get the specimen, sealed in the internal holder cavity, from the bench into preset gas-laden microscope.

We began our trials with inert gases, low pressures, and with microscope column valves closed. We then proceeded progressively to adjust conditions towards the operating limits of the microscope, carefully evaluating the microscope response during, and recovery after, each gas experiment. In Fig 9, we show measured pressure readings during the loading of the specimen, as well as an added illustration of the presumed pressure in the specimen holder chamber, prior to opening the chamber in the internal microscope ambient. First, we set up the microscope to the desired water vapor condition, verified the stability and noted the instrumental parameters (A-B). On the bench, we also loaded the specimen into the holder (P-Q). Given that microscope interlocks prevent the loading of specimen while the gas inlet valve is open, and while the pressure reading is above 0.06 kPa, we closed the inlet valve (C) and allowed the pressure to fall to release the interlock (D). We loaded the specimen, still enclosed in the internal holder chamber cavity at ∼1 atm, into the microscope (R), and quickly reopened the gas inlet (E) to re-establish the desired microscope specimen area pressure. When the target specimen pressure was reached (F), we opened the holder internal chamber, to allow the specimen to equilibrate to the microscope ambient (T). This specimen loading process took approximately 5 minutes. From T onward, the electron beam column valves could be opened and specimen imaging in the preset gas atmosphere, could be performed. After the experiment, the gas inlet supply was closed and the outlet pumping stages were sequentially opened up to recover the high vacuum condition, as shown from G-L.

After establishing the feasibility of this loading sequence, we performed brief trials with liquid water. For the specimen, 3 μL ultra pure liquid water was added to a (plasma-activated, hydrophilic) TEM grid, and the microscope was set up with 1.3 kPa water vapor. As mentioned previously, without a cooling holder, liquid water would be expected to evaporate quickly at room temperature at this partial pressure, but this trial was of value to evaluate the feasibility of the loading process. After loading as shown in Fig 9, we opened the column valves to inspect the specimen. Despite the sub-saturation condition, liquid water was briefly seen on the grid, and observed to evaporate under the electron beam, as shown the Supporting Information (S7 Fig). This basic test verifies that transfer of a specimen in a hydrated state is possible in principle; and illustrates that further experiments with an appropriate specimen holder hardware (with combined cooling and transfer capabilities) would be a worthwhile exercise. It seems possible that wet specimens or liquid water could be observed in a stable and controlled fashion, without the need for any enclosing windows. We elaborate more on the future possibilities for hydrated specimen experiments in the Discussion section.

### Microscope recovery after water exposure

**Fig 10:**
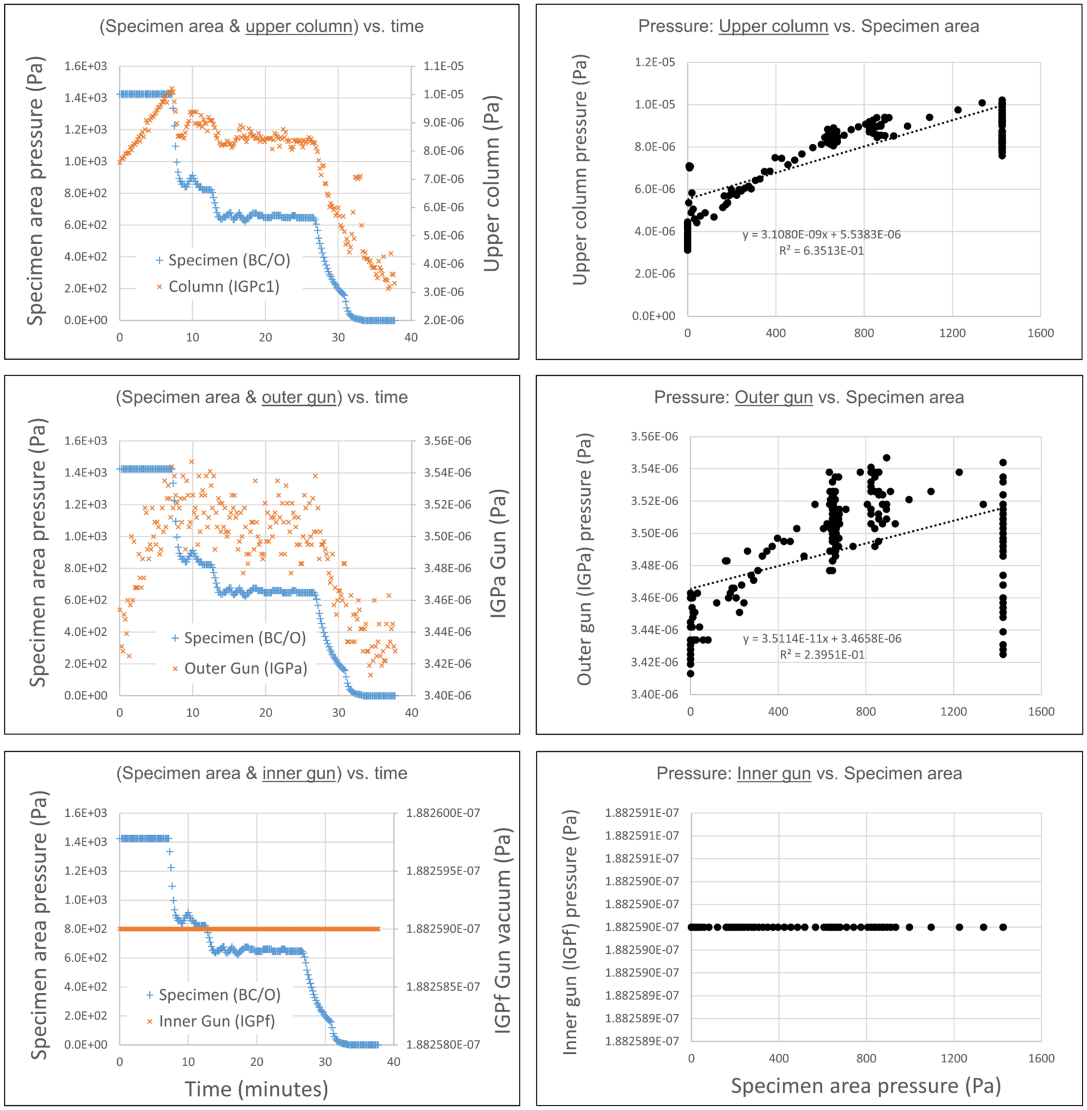
Upper column and electron gun vacuum levels during specimen area water vapor exposures (column valves open). As expected, the correlation between with specimen area pressure and the gun area pressure weakens closer to the electron gun. There was no detectable shift in inner gun vacuum readings, with maximum (kPa) levels of water vapor in the specimen area. The labels BC/O, IGPc1, IGPa and IGPf refer to the specific microscope hardware used.

While water vapor is an approved process gas for the utilized microscope hardware, nevertheless there is very little information in the public domain about the effect of water vapor exposures on the performance of the microscope, especially in the higher pressure regime explored in this work. With this in mind, we consider the monitoring of microscope performance, as a function of water vapor exposure, to be a necessary component of the experiments. First, we show the effect of water vapor introduced into the specimen area, upon pressure readings in the sensitive electron gun area. In Fig 10, we show pressure readings (acquired with microscope column valves open) in the upper region of the microscope in the vicinity of the electron gun, as a function of the water vapor pressure in the specimen area. The gun-area vacuum readings are all within an acceptable range, indicating that the differential pumping configuration is effective in protecting the sensitive gun components from the high specimen area pressure. Specifically, a supply of 1.4 kPa water vapor in the specimen area caused less than 90 μPa change in the upper column, less than 150 nPa change in gun accelerator region, and no measurable change in the gun emitter area (pressure readings from IGPc1, IGPa and IGPf, respectively, for those familiar with this particular microscope hardware). Note, however, that there is a steady increase in the upper column and outer gun reading when the specimen area is in a steady high pressure state (1.4 kPa, 0-8 minutes). This suggests that care should be taken to avoid extended periods with high pressures and column valves opened, and that the gun vacuum levels should be closely monitored at all times.

**Fig 11:**
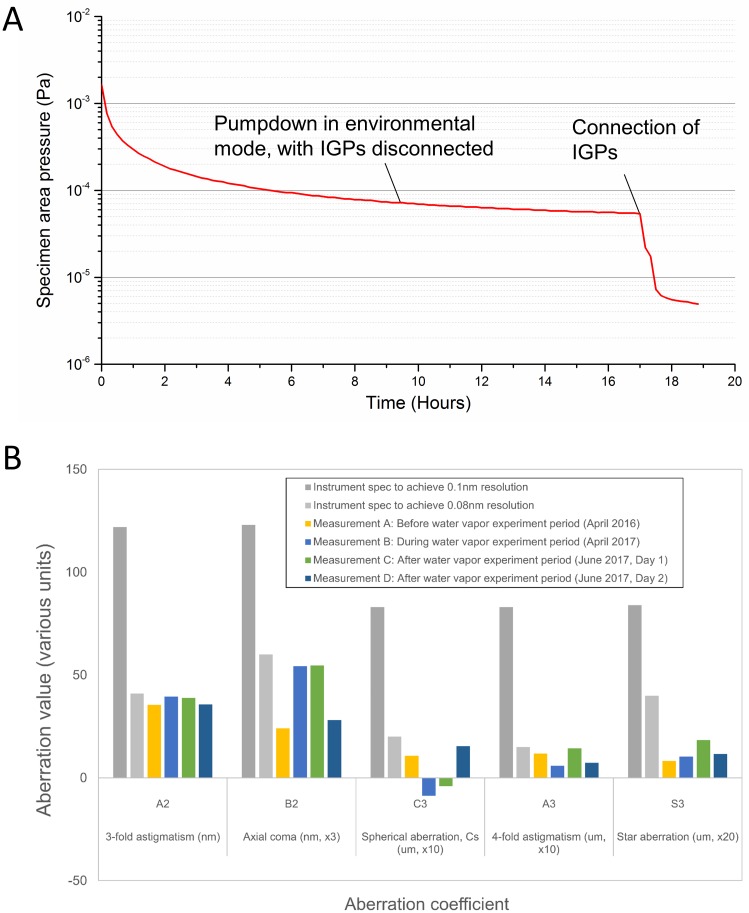
Post-water vapor vacuum logging and aberration measurement. A. After execution of water vapor experiments, the system was typically left pumping overnight, and had fully recovered to normal vacuum levels by the following morning. B. Electron optical aberrations, as measured using the C_s_-corrector hardware, showed no shift as a function of water vapor exposure.

In Fig 11A, we show the recovery of the specimen area vacuum after one day of water vapor experiments. At the end of the day’s experiments, the system was left to pump overnight in environmental mode (which has numerous high-throughput turbo-molecular pumps connected, but with the Ion Getter Pumps (IGPs) disconnected). The following morning, having reached a pressure reading on the order of 10^-5^ Pa, the system was returned to conventional high vacuum mode, with the IGPs connected. The pressure quickly reached the 10^-6^ Pa range. Note that liquid nitrogen was not supplied to the cold finger reservoir during recording of the pressure curves shown in Fig 11A, so even better vacuum levels could be attained if liquid nitrogen cooling were employed. It is clear that the vacuum system recovers to normal levels without difficulty.

Additionally, we have checked if there is any degradation in the electron optical imaging performance, by checking the measured optical aberrations during image correction, historically and in the timeframe of the water vapor experiments. Results are summarized in Fig 11B. As can be seen, aberration values corresponding to an achievable resolution of less than 0.1 nm could readily be attained in all cases. Within the measurement accuracy, no change or degradation in the optical performance could be discerned. Furthermore, at the corrected condition, there was no significant shift in the current drawn by any of the optical components in the spherical aberration corrector, before and after water vapor experiments, indicating that the optical environment is quite stable (S8 Fig).

## Discussion

These results demonstrate the capabilities for supply, characterization, imaging and specimen loading for a microscope laden with water vapor in the saturation pressure regime. We have directly characterized the resolution limits, and confirmed that biological specimen contrast and stability are not drastically degraded by the presence of water vapor. It seems that this is a promising research theme that is worthy of further investigation. It certainly seems feasible that liquid water could be maintained in the specimen area, with the appropriate microscope hardware. As noted in the Introduction, working with liquids in an open cell configuration could have obvious advantages in enabling dynamic studies, and also allow a large field of view and high tilt specimen imaging.

Beyond the confirmed results and obvious advantages, we would like to speculate more generally on the possibilities that this configuration may offer for life science microscopy. First, wet sample preparation is a central requirement for this kind of work, that we have not mentioned so far. There are a few different approaches that might be followed. Liquid solutions might be dispensed on a suitable TEM substrate (such as amorphous carbon, SiN, or graphene), with appropriate volume and surface hydrophilicity to allow electron transparency, although achieving the optimum conditions might be challenging. Alternatively, hydrated specimens might be imaged on a support grid (such as thin carbon or graphene), without an explicit encapsulating solvent (water) matrix. The first monolayers of water surrounding biomolecules, the so-called hydration shell [35], which heavily influence the structure and function of the molecule, could be explored directly with this configuration. Another configuration could be the suspension of filamentous specimens over holes in a support grid, and then their imaging in a hydrated state with no solid state background at all (such suspension of filamentous specimens has already been demonstrated in dry conditions (e.g. M13 phage [33], and DNA [36])). Finally, a more ambitious idea for native-state electron microscopy might be that of free-standing (suspended) liquid water. While this sounds rather difficult to achieve, recently Mizoguchi et al. [23] demonstrated preparation of free-standing liquid with 10nm thickness (using ionic liquids with low vapor pressure), and acquisition of high quality electron microscopy data. Given the control of the ambient water vapor partial pressure demonstrated in this work, this configuration might also be possible in water, at least in principle, and is an exciting prospect for the future.

Of relevance to all of these suggested approaches is that utilizing a water vapor ambient offers the potential to tune the sample preparation, “live” in the TEM. This is in sharp contrast to ice matrices or the windows of closed cells, which are prepared *ex situ* and cannot be adjusted in the microscope. This could potentially bring specimen optimization, widely acknowledged as the main limiting factor in life science electron microscopy [37], under direct observational control in the microscope. Indeed, the basic components for *in situ* sample preparation already exist, namely the ability to dispense controlled nanoscale volumes of bio-specimen solution [38, 39], and the ability to micro-position a supply needle and dispense materials inside an electron microscope [40]. With this work we demonstrate that a microscope ambient capable of sustaining such *in situ* liquid dispensation, while preserving sufficient imaging performance, is possible.

A critical topic in life science microscopy is radiolysis of the specimen, whose effect varies greatly with the microscopy configuration (e.g. accelerating voltage) and specimen properties (e.g. conductive or insulating nature) [41]. Aside from primary knock-on and ionization damage, the role of secondary chemical reactions, involving the generated ions and radicals, is perhaps even more critical. Some work has been published previously outlining possible chemical pathways for radiolytic damage in ice [42] and liquid water [43] specimens, although this is a very complex field that is still not well understood. Working with gas solvents provides a completely new testbed with which to explore the science of radiolysis. In particular, the fact that the solvent molecules are very sparse, and constantly being swept away and replaced by a fresh supply of new (neutral) gas molecules, is rather different from the conventional cryo- or closed cell-microscopy, and thus interaction of damaging radicals with the specimen may be studied, or even controlled. Whether or not the gas configuration is better than conventional techniques in terms of specimen radiolytic damage, at the very least, utilization of solvent gases should provide us with a fresh viewpoint on radiolytic processes.

The theory of image formation in a gas ambient [44] is also highly relevant to the current discussion, and in particular the central role played by the contrast variations of the support or solvent material. Under typical acquisition conditions in a gas ambient, each successive electron wave acts in a fashion similar to a strobe light, taking a snapshot of (effectively stationary) gas molecules. These gas molecules move to new positions between successive exposures (while the atoms of the solid-state specimen remain fixed), which causes the gas-related background to smooth out as the integrated micrograph is built up. In contrast, the fixed specimen contrast is obviously reinforced. This was directly demonstrated by Yoshida and Takeda [44], using simulations of carbon nanotubes in an ethanol gas ambient, and it would not require a big leap of imagination to see the similarities with filamentous virions in water vapor. Thus, using a gas as the effective specimen “solvent” may be quite a favorable approach, in comparison to solid encapsulants like ice or SiN windows, whose non-uniformities will cause an unavoidable background contrast. Furthermore, in our EELS studies we noted that, in TEM mode a significant proportion of the energy loss electrons, which disappeared from the zero-loss peak in the presence of water vapor, were not detected in the energy loss region of the spectrum, and therefore must have been absorbed by the apertures of the system (S3 Appendix). This was borne out by reduced values of measured electron beam currents as a function of water vapor pressure in TEM mode, indicating that some electrons are being scattered out of the optical path. Such energy loss electrons, considering their different wavelength, would be focused incorrectly and degrade the contrast in the image plane. It would seem that in scattering by a gas column, a large proportion of these energy loss electrons are self-filtered by the system apertures and do not contribute to the final image. This is another indication that utilizing a gas solvent may have some advantages for image contrast, and at a minimum, provides incentive for further investigations.

Finally, dynamic high resolution electron microscopy and spectroscopy in water vapor could yield valuable insight into basic properties of water itself, which despite its ubiquitous nature still remains mysterious and continues to reveal attributes that surprise us [45]. In particular, nanoscale volumes of water will undoubtedly show quite different behaviors than the corresponding bulk, with direct implications for processes like nucleation of water droplets (e.g. in atmospheric science) and wet industrial processes (e.g. in nanodevice patterning in the semiconductor industry). It is hoped that the preliminary studies presented in this paper will stimulate further work in high resolution microscopy of liquid and vapor phase water.

## Conclusion

We have demonstrated the supply and control of water vapor, in the pressure range which corresponds to saturation conditions for specimen temperatures of 273-290 K, in an open cell transmission electron microscope. The effective thickness of the gas ambient was evaluated using EELS, and compares favorably to existing cryo- and closed-cell approaches. Microscope resolution remained in the ≤ 2Å regime for the highest pressures applied, and no significant contrast loss or specimen instability was noticed for biological specimens imaged in water vapor. These are encouraging first results. To advance this topic, recommended next steps are to repeat the experiments in conjunction with specimen temperature control (cooling transfer holder); and to use a TEM with a hardware configuration optimized for sensitive, low-dose microscopy. It remains to be demonstrated if this scheme can offer genuine advantages over conventional cryo-microscopy, or “closed cell” liquid and vapor phase TEM; but certainly the feasibility of atomic resolution imaging in saturated water vapor conditions has been demonstrated, and the quantitative study of water vapor in the transmission electron microscope opens up a host of exciting research opportunities.

## Supporting information

S1 Appendix Definition of the variables used in Eqs 1–3. Strike rate, escape rate, and mass loss rate.
(DOCX)

S2 Appendix Further experimental details. Full information on settings for EELS acquisitions, HRTEM resolution tests, and biological specimen image acquisition.
(DOCX)

S3 Appendix EELS effective thickness evaluations. Further information and analysis of gas effective thickness (t/λ), as a function of pressure, using different calculation methodologies and using data acquired in TEM and STEM mode.
(DOCX)

S4 Appendix Specimen area pressure estimates derived from EELS scattering data. Although all the required parameters are not known precisely, an approximate estimate is possible, based on literature values, and common-sense estimates of equipment parameters. These calculations yield pressure values which are consistent with those reported by the system-level pressure gauge. The most important message from this exercise is that there is no indication that the actual pressure at the specimen area is lower than the value reported by the system-level pressure gauge.
(DOCX)

S1 Dataset Reference (native format) EEL spectra from pure water vapor, at various pressures. This could be used for MLLS fitting of water vapor content in gas mixtures. These were acquired in STEM mode, with exact settings included in S2 Appendix.
(ZIP)

S2 Dataset Line profiles through individual M13 virions. Micrographs showing the locations of the extracted profiles, as well as the individual profiles themselves, are included.
(ZIP)

S1 Fig Initial RGA measurement, showing significant residual atmospheric gases. This validates the RGA sensitivity to those gases, and corroborates the purity of water vapor shown in later measurements.
(JPG)

S2 Fig Reference RGA spectra from 6N purity hydrogen, oxygen and nitrogen. Note that trace water vapor is present in the hydrogen spectrum only (as the same supply line was used previously for water vapor delivery).
(JPG)

S3 Fig Variation of RGA ion currents as a function of inert gas pressure. Note that the relationships between pressures and RGA ion currents were not linear. We attribute this to a significant time lag between equilibration of pressure conditions in the specimen area and at the RGA. The RGA operating pressure was many orders of magnitude below the specimen area pressure (∼10^−4^ Pa vs. ∼10^3^ Pa); therefore, an almost closed needle valve was used to minimize pumping conductance and “step down” the pressure experienced by the RGA. The specimen area pressure was adjusted continuously, and stabilization time is not allocated at each pressure. Thus, it is reasonable that the relationship with the pressure gauge and RGA readings is not linear.
(JPG)

S4 Fig Evaluation of the synchronicity and linearity between pressure gauge readings and EELS measurements. The microscope column valve open/close event logging was used to explicitly synchronize the data sets acquired from these completely independent hardwares. The responses to changes in gas supply conditions are simultaneous, indicating a uniform gas ambient and reliable measurement values.
(JPG)

S5 Fig HRTEM resolution at different beam currents. Higher beam currents degrade resolution, as reported previously by numerous authors for other gas species.
(JPG)

S6 Fig Specimen contamination under simultaneous electron beam and water vapor exposure. Deliberately excessive exposures were applied, to reveal the limiting damage mechanism in water/electron beam experiments.
(TIF)

S7 Fig Transfer feasibility for wet specimen. A wet sample (3 ul ultra-pure water on a standard grid) was successfully transferred into the gas-laden microscope, without exposure to intermediate high vacuum steps. Liquid water was observed evaporating under the electron beam.
(JPG)

S8 Fig Electrical current drawn by electron optical components in spherical aberration corrector, before and after water vapor experiments. There is no discernible shift in the current drawn by any of the components, at the corrected condition.
(JPG)

S1 Video Image stack acquired through 1.3 kPa water vapor, 10kx. While the magnification and pixel sampling are quite low, it is already evident that the sample is spatially and structurally quite stable, under simultaneous electron beam and water exposure. A high contrast defect feature was deliberately included to allow accurate specimen position tracking.
(WMV)

S2 Video Image stack acquired through 1.3 kPa water vapor, 43kx. Even after substantial stage movement to inspect different areas, the stage stabilizes quite quickly. Overall, the stability seems comparable to conventional, high vacuum microscopy, and does not seem to be a limiting factor in any way.
(WMV)
