# Supplementary material for: Water without windows: Evaluating the performance of open cell transmission electron microscopy under saturated water vapor conditions, and assessing its potential for microscopy of hydrated biological specimens
Source: PLoS One. 2017 Nov 3;12(11):e0186899. doi: 10.1371/journal.pone.0186899 (PMC5669482; doi:10.1371/journal.pone.0186899)
Supplement: S1 Appendix — Strike rate, escape rate, and mass loss rate. (DOCX) [file pone.0186899.s001.docx]

**S1 Appendix – Definition of constants in Eq. 1-3**

n_v_ = number density of water molecules in the vapor

m = mass of water molecule

k = Boltzmann’s constant

T_v_ = temperature of water vapor

u = velocity of water molecule (dummy variable in the integration)

n_l_ = number density of water molecules in the liquid

T_l_ = temperature of the liquid water

ϵ = latent heat of vaporization of a single molecule

P_w_ = partial pressure of water

X(T_l_) = saturated vapor pressure at temperature T_l_, when T_l_ = T_v_.

m_E_ = mass change due to evaporation

ρ_w_ = density of liquid water
