# Supplementary material for: Water without windows: Evaluating the performance of open cell transmission electron microscopy under saturated water vapor conditions, and assessing its potential for microscopy of hydrated biological specimens
Source: PLoS One. 2017 Nov 3;12(11):e0186899. doi: 10.1371/journal.pone.0186899 (PMC5669482; doi:10.1371/journal.pone.0186899)
Supplement: S2 Appendix — Full information on settings for EELS acquisitions, HRTEM resolution tests, and biological specimen image acquisition. (DOCX) [file pone.0186899.s002.docx]

**S2 Appendix – Details on acquisition settings for EELS, HRTEM resolution tests and bio-specimen image acquisition.**

EELS acquisitions were acquired using a post-column Gatan Quantum 966 spectrometer and the Digital Micrograph software package. Spectra were acquired in both STEM mode (with a very well defined collection angle geometry [Cassidy2017]) and TEM mode (for correlation with TEM imaging experiments). In STEM mode, the following settings were used: convergence angle 10 mrad, collection angle 40.5 mrad, acquisition time 1-200 μs/spectrum, energy dispersion .05 eV/channel. The Dual-EELS function was utilized to simultaneously capture the zero-less and low-loss spectra with different integration times (1ms and 200ms, respectively), and the Spectrum Imaging function was utilized to build up two-dimensional plots of EELS counts as a function of both time and energy loss.

Typical settings for HRTEM resolution tests were: magnification 380kx, electron dose rate: 400e/A^2^/s. Aberration correction was performed under UHV conditions prior to executing water vapor experiments, with an applied tilt angle of 24 mrad and measured aberration values of: 39nm (3-fold astigmatism), 18nm (coma), -875um (spherical aberration), 582nm (4-fold astigmatism), and 515nm (star aberration). Respective confidence interval values were 53.6nm, 30.9nm, 2.6um, 1.5um, and 0.9um. Phase plate values, excluding defocus and 2-fold astigmatism terms, were 19mrad for sub- $\pi$/4 phase shift range (limited by coma), and 59mrad (limited by star aberration) for 12 $\pi$ range. During micrograph acquisitions, 2-fold astigmatism was corrected continuously, and defocus was chosen such that the first passband of the CTF corresponded with the first lattice ring from crystalline Au (as reported previously [Jinschek2012]).

Bio-specimen contrast evaluation was also performed in TEM mode. Images were acquired with magnifications of 10kx, 43kx and 115kx, and approximate electron dose rates of ≤ 1, 10, and 70 e/Å^2^/s. The utilized hardware was not optimized for low-dose microscopy, so these images are not intended to be competitive with state-of-the-art life science EM. Rather, these studies are useful for comparison of image quality between the gas and non-gas cases. The specimen was M13 phage, applied to homemade amorphous carbon supports, with thickness of approximately 20nm.

References:

- Cassidy C, Dhar A, Shintake T. Determination of the mean inner potential of cadmium telluride via electron holography. Applied Physics Letters. 2017;110(16503):1-5.
- Jinschek JR, Helveg S. Image resolution and sensitivity in an environmental transmission electron microscope. Micron. 2012;43:1156-1168.
