# Supplementary material for: Water without windows: Evaluating the performance of open cell transmission electron microscopy under saturated water vapor conditions, and assessing its potential for microscopy of hydrated biological specimens
Source: PLoS One. 2017 Nov 3;12(11):e0186899. doi: 10.1371/journal.pone.0186899 (PMC5669482; doi:10.1371/journal.pone.0186899)
Supplement: S3 Appendix — Further information and analysis of gas effective thickness (t/λ), as a function of pressure, using different calculation methodologies and using data acquired in TEM and STEM mode. (DOCX) [file pone.0186899.s003.docx]

S6 Appendix – EELS scattering from gas column

- The standard definition of mean free path [S1] is:

$\frac{t}{\lambda}=ln\left( \frac{\int I_{T}dE}{\int I_{ZL}dE} \right)$ (1)

where I_T_ refers to the total incident electron intensity, and I_ZL_ refers to the unscattered electrons (i.e. detected in the zero-loss peak of the EELS spectrum).

- I_T_ from vacuum zero-loss peak (A)

I_T_ can be directly obtained by measuring the zero-loss peak in vacuum (in the absence of any specimen), as I_T_ = I_ZL(vac)_, which leads to the following expression for mean free path:

$\frac{t}{\lambda}=ln\left( \frac{\int I_{ZL(Vac)}dE}{\int I_{ZL}dE} \right)$

For the case that the object is a gas of pressure P, we have:

$\left( \frac{t}{\lambda} \right)_{P}=ln\left( \frac{\int I_{ZL(Vac)}dE}{\int I_{ZL\left( P \right)}dE} \right)$ (2)

The benefit of this method is that the zero-loss peak measurement is relatively straightforward, and typically has high measurement accuracy owing to high zero-loss count rates. However, a feature of this method is that it makes no distinction regarding the scattering processes which caused the loss of electrons – any missing electrons from the incident beam will be incorporated into the mean free path calculation, irrespective of the scattering mechanism. For example, high angle *elastically* scattered electrons, if intercepted by the system apertures, will be incorporated into the calculated mean free path value. This method makes no distinction between electrons that did not propagate through the optical system, and energy-loss electrons that were successfully captured by the detection system.

- I_T_ from energy loss counts (B)

I_T_ can also be obtained by measuring the sum of the zero-loss and loss signals, in the presence of the specimen, as I_T_ = I_ZL(specimen)_ + I_L (specimen)_, which leads to the following expression for mean free path:

$\frac{t}{\lambda}=ln\left( \frac{\int I_{ZL}+I_{L}dE}{\int I_{ZL}dE} \right)$ (3)

For the case that the object is a gas of pressure P, we have:

$\left( \frac{t}{\lambda} \right)_{P}=ln\left( \frac{\int I_{ZL\left( P \right)+}I_{L(P)}dE}{\int I_{ZL\left( P \right)}dE} \right)$ (4)

This is generally a commonly employed and convenient method, and has the advantage that a reference zero-loss peak in vacuum is not needed. This method relies upon measurement of the energy loss counts (I_L_). This may be advantageous, if only the specific contribution from inelastically scattered electrons is desired. However, the efficiency of detection of the energy loss electrons depends upon the collection angle, so a disadvantage is that the reported value is very sensitive to instrumental parameters. Furthermore, the count levels will typically be quite low compared to the zero-loss counts, so the measurement signal-to-noise will typically be poorer than the vacuum zero-loss peak case.

- If the measurement environment is a closed system, such that all elastically scattered electrons are successfully detected in the zero-loss peak, and all inelastically scattered electrons are successfully detected in the loss region, and no electrons are lost to the system apertures, then the calculations described above will produce identical values for mean free path.

For the case of EELS measurements of water vapor, we have an unusual situation in that we have a gas “object” which extends over several mm, far from the usual specimen focal plane. As such, the collection geometry is rather ill-defined in comparison to normal EELS measurements of a specimen at the objective lens focal plane. With this in mind, we have evaluated the mean free path using both of the methodologies introduced above. We have also performed the measurements in both STEM mode (in a standard configuration, with a very well-characterized high collection angle geometry [S2]), and in TEM mode (for correlation with TEM image acquisitions, but with poorly understood collection angle geometry). The results are summarized in Figure S6a below.


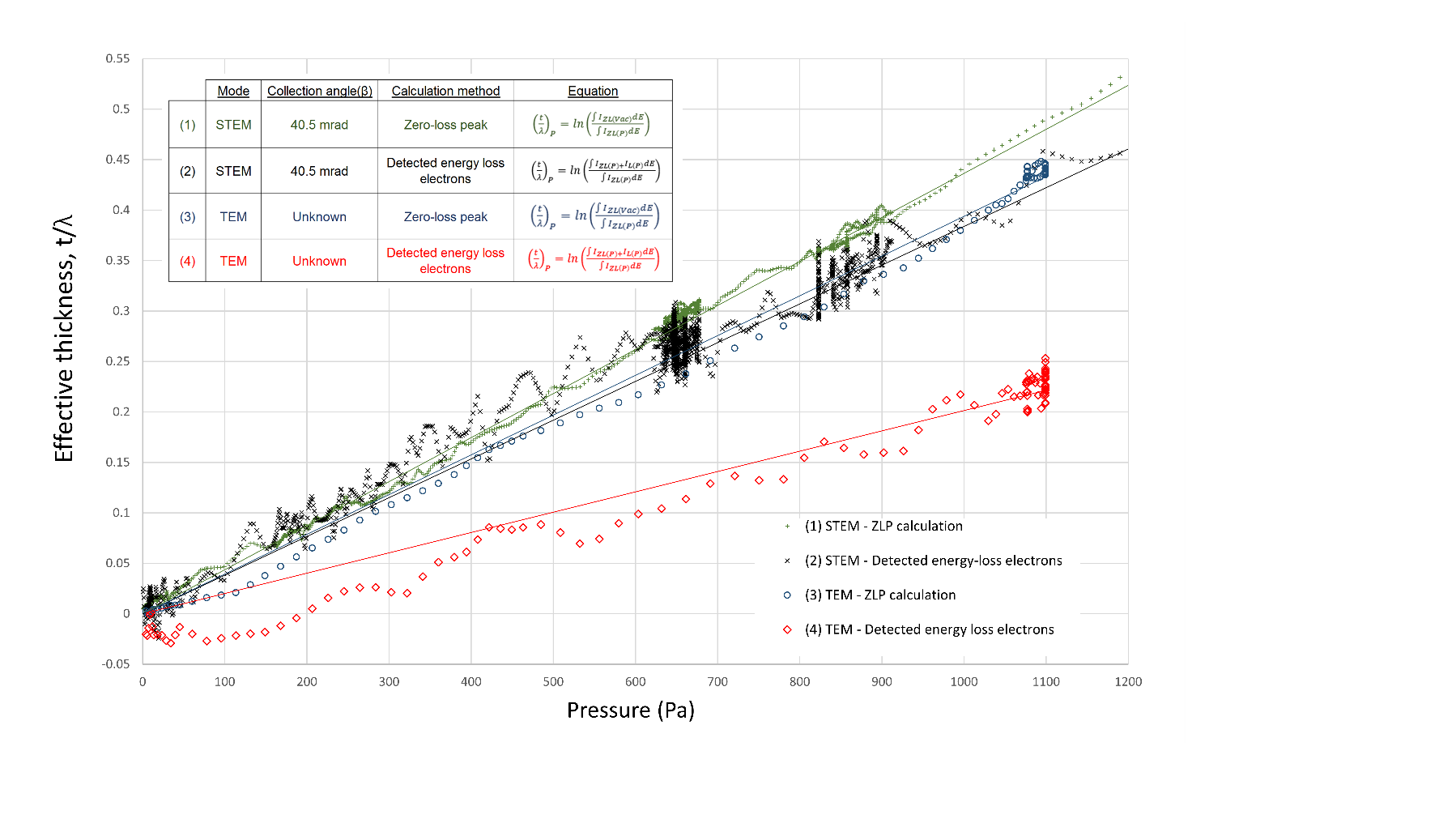


Fig. S6a – Mean free path vs. water vapor pressure, for various electron beam configurations and calculation methods.

Based upon Fig. S6a, we can make the following observations:

- By comparing curves 1 & 2, we can conclude that in STEM mode, almost all (90%) of the scattered electrons, lost from the zero-loss peak, are successfully captured by the detection system. This also indicates that absorption of elastically-scattered electrons is not a major contributor in this configuration ( < 10%). It is most likely that the 10% discrepancy relates to the poor SNR in the loss-electron signal, rather than genuine differences in electron scattering mechanisms.
- By comparing curves 1 & 3, we can see that we have good agreement between the measured mean free paths in TEM and STEM mode, if only the zero-loss peak data is considered for the calculation. This is expected, given that the zero-loss peak does not show such sensitive dependence on collection angle and other instrumental parameters.
- By comparing curves 3 & 4, we can conclude that a significant proportion of inelastically scattered electrons are absorbed by the system apertures in TEM mode, and are not detected by the detection system. This may be a favorable situation in TEM imaging, as inelastically scattered electrons will be incorrectly focused and would only contribute unwanted background to the detected image.

In the main article, we have reported Curve (1), for the following reasons:

- It is the best-defined measurement geometry, with best signal-to-noise ratio.
- The scattering is predominantly inelastic, without major contribution from “scattering absorption” of elastically scattered electrons.
- Curve 1 reports the highest value of effective thickness, so we are erring on the conservative side in our reported values, when comparing against other scenarios like cryo-EM and closed cells.

References:

S1. R. F. Egerton, Electron Energy Loss Spectroscopy in the Electron Microscope, 3rd Ed. (Springer, 2011).

S2. Cassidy C, Dhar A, Shintake T. Determination of the mean inner potential of cadmium telluride via electron holography. Applied Physics Letters. 2017;110(16503):1-5.
