# Supplementary material for: Water without windows: Evaluating the performance of open cell transmission electron microscopy under saturated water vapor conditions, and assessing its potential for microscopy of hydrated biological specimens
Source: PLoS One. 2017 Nov 3;12(11):e0186899. doi: 10.1371/journal.pone.0186899 (PMC5669482; doi:10.1371/journal.pone.0186899)
Supplement: S4 Appendix — Although all the required parameters are not known precisely, an approximate estimate is possible, based on literature values, and common-sense estimates of equipment parameters. These calculations yield pressure values which are consistent with those reported by the system-level pressure gauge. The most important message from this exercise is that there is no indication that the actual pressure at the specimen area is lower than the value reported by the system-level pressure gauge. (DOCX) [file pone.0186899.s004.docx]

**Using EELS scattering data to deduce water vapor pressure in the specimen area**

We have reported EELS measurements of effective thickness (t/λ) of water vapor, as a function of pressure. We can utilize this data to deduce how many water molecules the electron beam encountered as it travelled from emitter to detector, and with some estimates on the dimensions of the differential stages (t), can convert these values to pressure values.

There are two input parameters that are needed:

1. The scattering cross-section of a water molecule for 300kV incident electrons
2. Knowledge, or estimates of the spatial distribution of the water vapor molecules. In this case, this means the distribution of the molecules along the z-direction. In practice, it means the dimensions and pressures in the various stages of the differential pumping system.

**1) Individual water molecules scattering cross-section for 300kV electrons**

1. Theoretical value: From [Munoz], utilizing Eq. (1) and Table II, leads to the range

$\sigma_{inel}=\left( 5.8-10.8 \right) x {10}^{-23} m^{2}$

for single water molecules and 300kV electrons.

1. Experimental value (derived from EELS ice measurement at 300kV).

Experimental values for water vapor at 300kV are not available, so we have used ice as the closest available approximation. A drawback is that, presumably the scattering cross-section of a water molecule will be affected somewhat by whether the molecule is bonded in the solid state, or free in a vapor. A strong point, however, is that the macroscopic density (i.e. number of molecules) is known, and the mean free path measurement was performed using EELS at 300kV, so is directly comparable to our measurement. At a minimum, this calculation allows us to check if the theoretical values, mentioned above, are of the right order of magnitude.

From [Vulovic], $\lambda_{300kV(ice, EELS)}=330nm.$

From [Williams and Carter],

$\sigma_{molecule}=\frac{1}{\left( \frac{N}{V} \right)\lambda}=\frac{1}{N^{'}\lambda}=\frac{m}{\rho\lambda}$ (1)

where N/V is the number of molecules per unit volume, m is the water molecular mass, and ρ is the macroscopic density.

Setting ρ_ice_ = 0.93 x 10^3^ kg/m^3^ [Leapman], and λ = 330nm [Vulovics], leads to

$$\sigma_{molecule}=9.7427 x {10}^{-23}m^{2}$$

This falls within the range of the theoretical predictions from [Munoz], and is valuable as the experimental measurement method is EELS at 300kV, which matches our current work. Note that ice was used as values of EELS effective thickness for vapor have not been published.

**2) Deriving an expression for pressure, in terms of σ (previous section), and t/λ (available from experimental measurements).**

We assume that the ideal gas law holds (given that we are in a relatively low pressure regime).

Firstly, we can easily express the measured effective thickness, t/λ, in terms of the number of molecules encountered by the electron beam. This is best expressed as an areal density, n_A_, of water vapor molecules (as the area defined by the electron beam can be adjusted arbitrarily, and the location of the molecules along the z-direction is unspecified). For convenience, we define $t_{eff}= \frac{t}{\lambda}$ and substitute into $\frac{N}{V}=\frac{1}{\lambda\sigma}$ from Eq. (1). This leads to:

$n_{A}=\frac{t_{eff}}{\sigma}$ (2)

To express pressure, P, in terms of mean free path and scattering cross-section, we can substitute $\frac{N}{V}=\frac{1}{\lambda\sigma}$ into the ideal gas law, which leads directly to:

$P= \frac{k_{B}T}{\lambda\sigma}$. (3)

where k_B_ is the Boltzmann constant, and T is the temperature (assumed 298 K). It is actually (t/λ) which is known from the experimental measurements, rather than λ, so we again use $t_{eff}= \frac{t}{\lambda}$ and substitute:

$P= \frac{{t_{eff}k}_{B}T}{t\sigma}$ (4)

We do not know the water vapor vertical distribution through the pumping stages of the microscope, and the exact dimensions of those pumping stages are not known (i.e. we do not know “t” in Eq. (3)). Thus, we cannot progress further with definite calculations. However, we are interested in evaluating if the EELS scattering measurements corroborate the nominal pressure readings, and in particular, are keen to rule out the case that the real pressure at the specimen area might actually be lower than the value reported by the adjacent capacitive gauge. Thus, we proceed with some approximate calculations, based on reasonable assumptions related to the known attributes of the experimental hardware.

**3) Further calculations, assuming that 100% of the water vapor is confined to the specimen area**

So, to progress further, we will have to make some assumptions. Firstly, we consider the case that all of the gas is confined in the central pumping stage. This is obviously not true, in that there must be some finite gas leakage to the outer stages, but it serves as the limiting case, and has the benefit that, in this case we know the individual P_0_ and t_0_ values. The configuration is summarized in the simple sketch below:


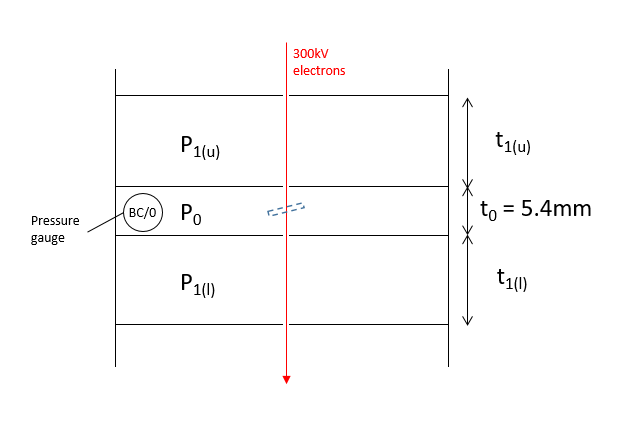


*Fig. S1 – Sketch of differential pumping stages.*

Using Eq. (4), and setting t = 5.4mm, we get the following plot:


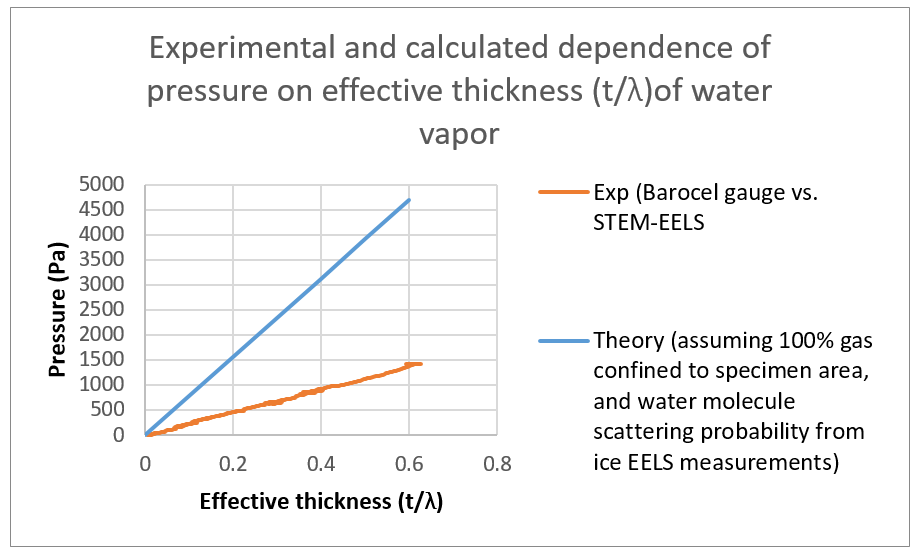


*Fig. S2 – Calculated relationship between effective thickness (t/λ), as measured by EELS, and water vapor pressure (assuming that all gas is strictly confined to the central pumping stage (blue curve). Experimental data (Curve 1 from Appendix 6), is added for convenience (orange curve). The calculated values (blue), derived from EELS, predict a much higher specimen area pressure than that reported by the pressure gauge (orange). This is reasonable, given that we have artificially condensed all the gas into the central stage, when in reality, there must be some finite gas pressure in the outer stages. These calculated values, derived from EELS data, suggest that it is unlikely that the capacitance gauge over-reports the actual specimen area pressure.*

**4) Estimates regarding gas pressure in the outer stages**

While we do not know the pressure distribution in the outer pumping stages, nor their dimensions, we can nevertheless perform some calculations, using the known parameters of the central (specimen) stage, and measured EELS data, as constraints. It is of interest to sanity-check the calculation methodology, by evaluating if the measured (gauge) and calculated (derived from EELS) pressures can be made to agree, by finding reasonable combinations of outer stage pressures and lengths.

Referring to the parameters introduced in Fig. S1, we can derive an expression for the pressure in the outer stage (P_1_), in terms of the effective length of the outer stage (t_1_):

$P_{1}=\left( \frac{1}{t_{1}} \right)\left( \frac{k_{B}T}{\sigma} \right)\left[ \left( \frac{t}{\lambda} \right)_{eff}-\left( \frac{\sigma}{k_{B}T} \right)\left( t_{0}P_{0} \right) \right]$ (4)

After inserting known values (t_0_ = 5.4mm; (t/λ)_eff_ = 0.6, P_0_ = 1400 Pa), we can plot the combinations of t_1_ and P_1_ which satisfy the experimental measurements (in this case, (t/λ)_eff_ = 0.6, P_0_ = 1400 Pa).


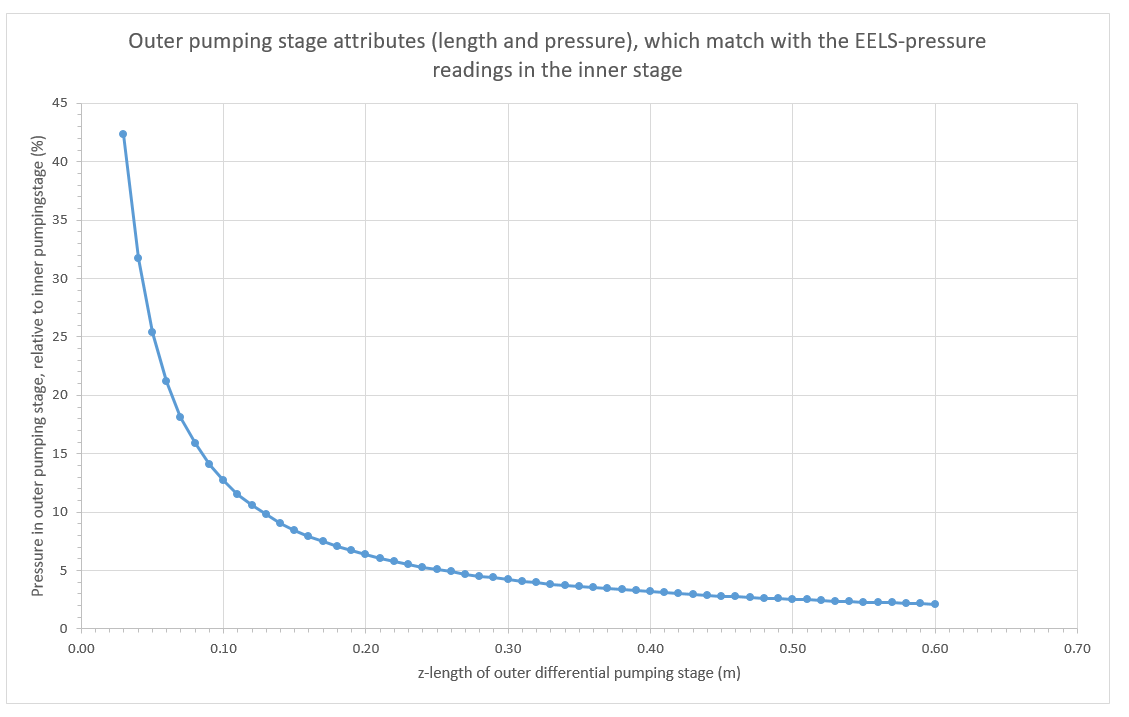


*Fig. S3 – Combinations of outer stage effective thickness and pressure, that result in agreement between measured (gauge) and calculated (from EELS scattering) pressure values. For example, the combination of 13cm outer stage length, with a pressure level that is 10% of the inner stage value, results in agreement between measured and calculated values; and is experimentally reasonable.*

In Fig. S3, the combinations of pressures and thicknesses in the outer stage, which result in agreement between measured (by gauge) and calculated (from EELS) pressure readings, are shown. Outer stage pressure is expressed as a percentage of the inner stage value. Without internal drawings of the microscope, we cannot make definitive conclusions, but the observed combinations seem experimentally reasonable. For example, if the outer pumping stage were 13cm long, and contained 10% of the central stage pressure, then the gauge measurements and EELS calculations would be mutually consistent.

As noted at the outset, our main goal with these calculations and estimates was to asses if we might be over-reporting the actual pressure at the specimen area. **These calculations consistently produce higher pressure values than those reported by the system pressure gauge, and thus it would seem highly unlikely that we are over-reporting the actual pressure at the specimen area**.

References:

- A. Muñoz, J. C. Oller, F. Blanco, J. D. Gorfinkiel, P. Limão-Vieira, and G. García, Electron-scattering cross sections and stopping powers in H2O, PHYSICAL REVIEW A 76, 052707 (2007)

- Vulovic M, Ravelli RBG, van Vliet LJ, Koster AJ, Lazic I, Lucken U, et al. Image formation modeling in cryo-electron microscopy. Journal of Structural Biology. 2013;183:19-32.

# - D. Williams, C. Barry Carter, Transmission Electron Microscopy: A Textbook for Materials Science, Springer (2009).

- Leapman RD, Sun S. Cryo-electron energy loss spectroscopy: observations on vitrified hydrated specimens and radiation damage. Ultramicroscopy. 1995;59:71-79.
