# Supplementary figures and images for: Water without windows: Evaluating the performance of open cell transmission electron microscopy under saturated water vapor conditions, and assessing its potential for microscopy of hydrated biological specimens
Source: PLoS One. 2017 Nov 3;12(11):e0186899. doi: 10.1371/journal.pone.0186899 (PMC5669482; doi:10.1371/journal.pone.0186899)

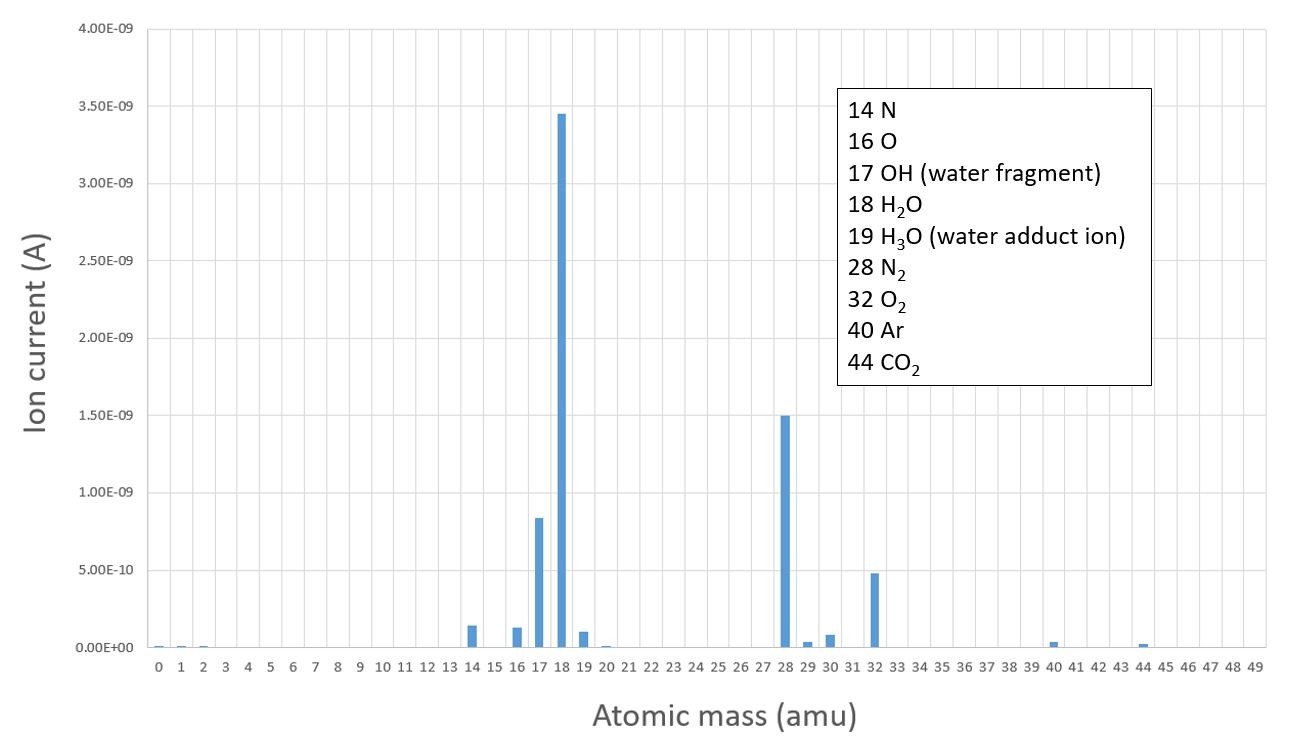

Supplement: S1 Fig — This validates the RGA sensitivity to those gases, and corroborates the purity of water vapor shown in later measurements. (JPG) [file pone.0186899.s007.JPG]

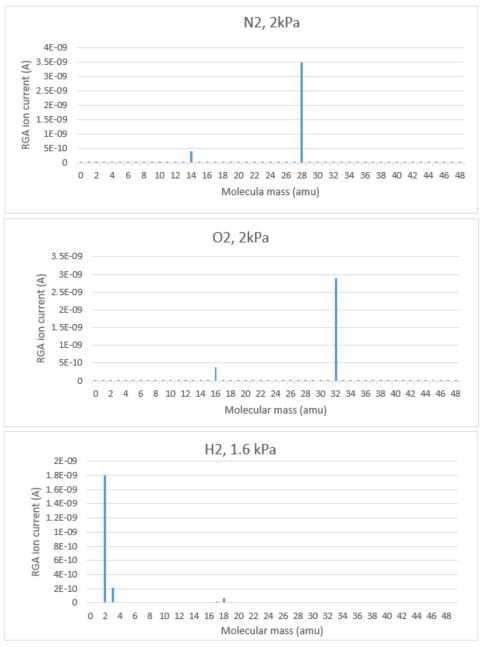

Supplement: S2 Fig — Note that trace water vapor is present in the hydrogen spectrum only (as the same supply line was used previously for water vapor delivery). (JPG) [file pone.0186899.s008.JPG]

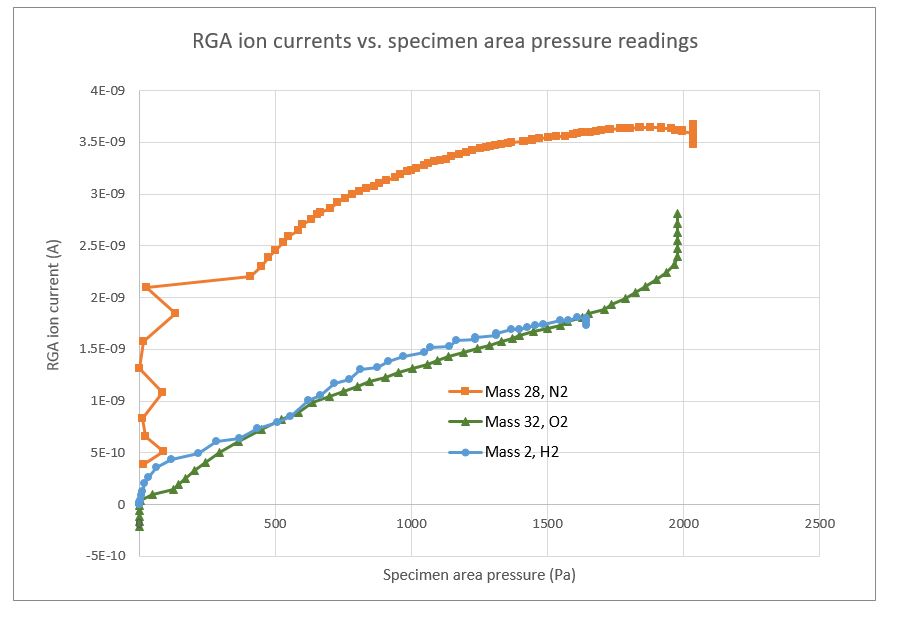

Supplement: S3 Fig — Note that the relationships between pressures and RGA ion currents were not linear. We attribute this to a significant time lag between equilibration of pressure conditions in the specimen area and at the RGA. The RGA operating pressure was many orders of magnitude below the specimen area pressure (∼10−4 Pa vs. ∼103 Pa); therefore, an almost closed needle valve was used to minimize pumping conductance and “step down” the pressure experienced by the RGA. The specimen area pressure was adjusted continuously, and stabilization time is not allocated at each pressure. Thus, it is reasonable that the relationship with the pressure gauge and RGA readings is not linear. (JPG) [file pone.0186899.s009.JPG]

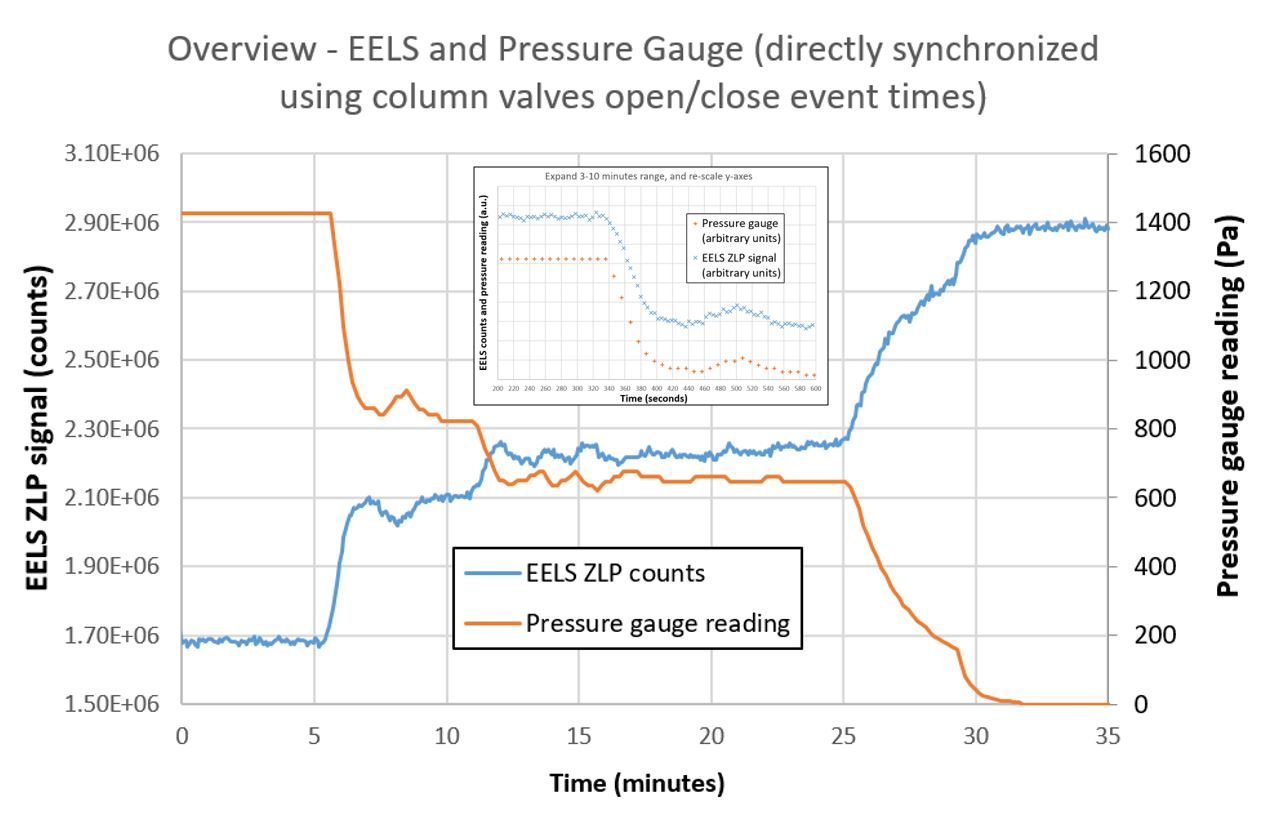

Supplement: S4 Fig — The microscope column valve open/close event logging was used to explicitly synchronize the data sets acquired from these completely independent hardwares. The responses to changes in gas supply conditions are simultaneous, indicating a uniform gas ambient and reliable measurement values. (JPG) [file pone.0186899.s010.JPG]

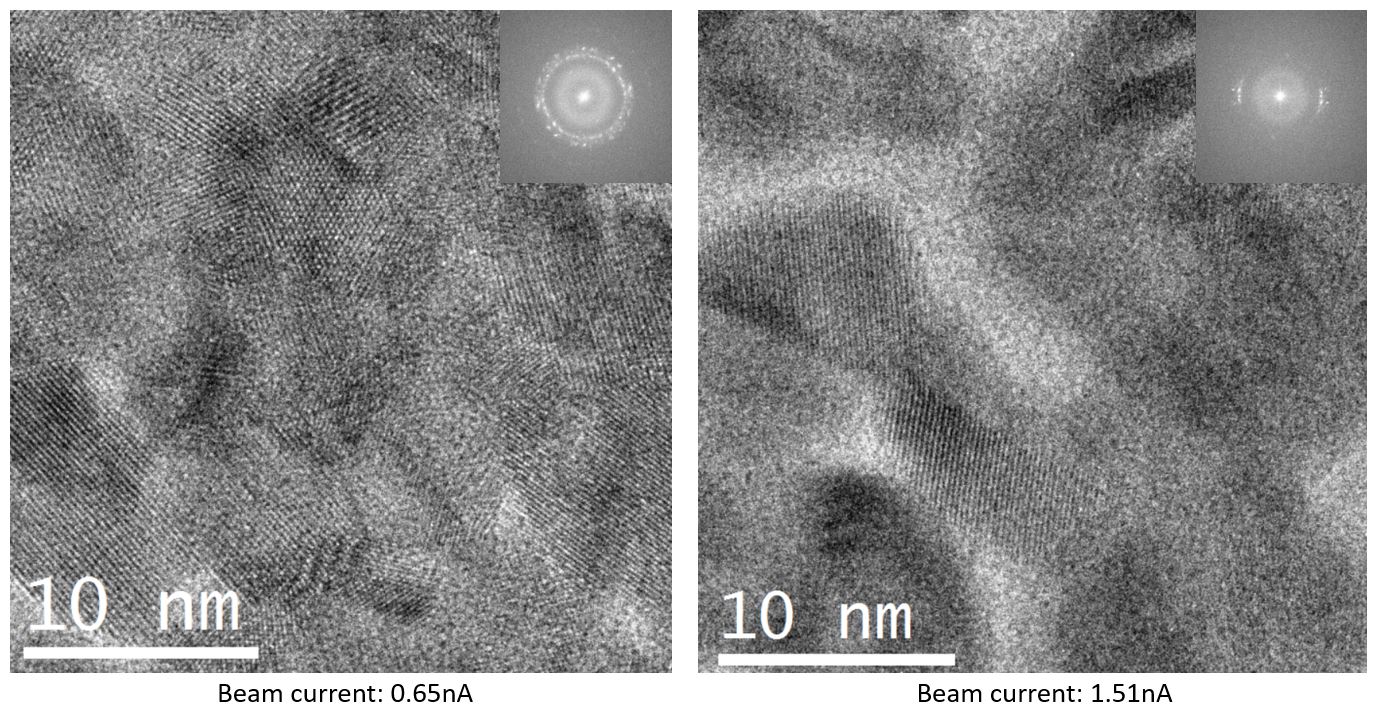

Supplement: S5 Fig — Higher beam currents degrade resolution, as reported previously by numerous authors for other gas species. (JPG) [file pone.0186899.s011.JPG]

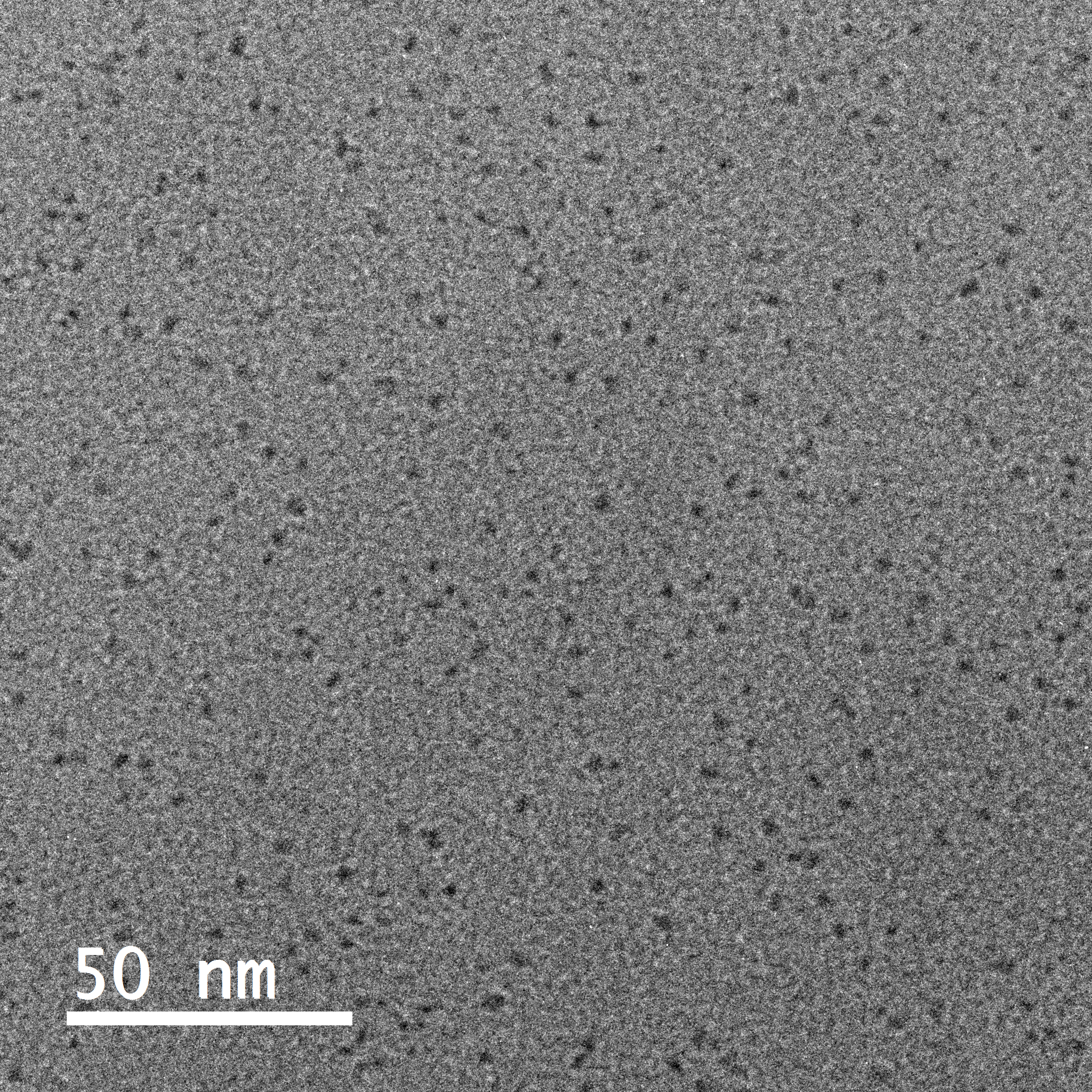

Supplement: S6 Fig — Deliberately excessive exposures were applied, to reveal the limiting damage mechanism in water/electron beam experiments. (TIF) [file pone.0186899.s012.tif]

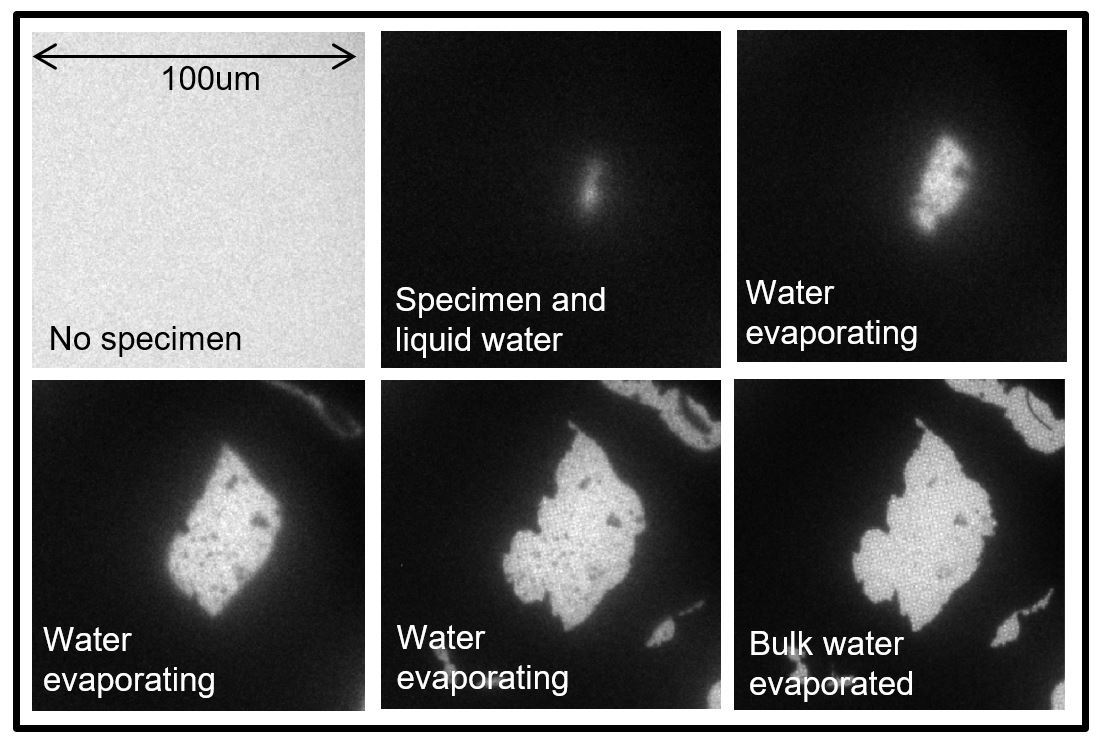

Supplement: S7 Fig — A wet sample (3 ul ultra-pure water on a standard grid) was successfully transferred into the gas-laden microscope, without exposure to intermediate high vacuum steps. Liquid water was observed evaporating under the electron beam. (JPG) [file pone.0186899.s013.JPG]

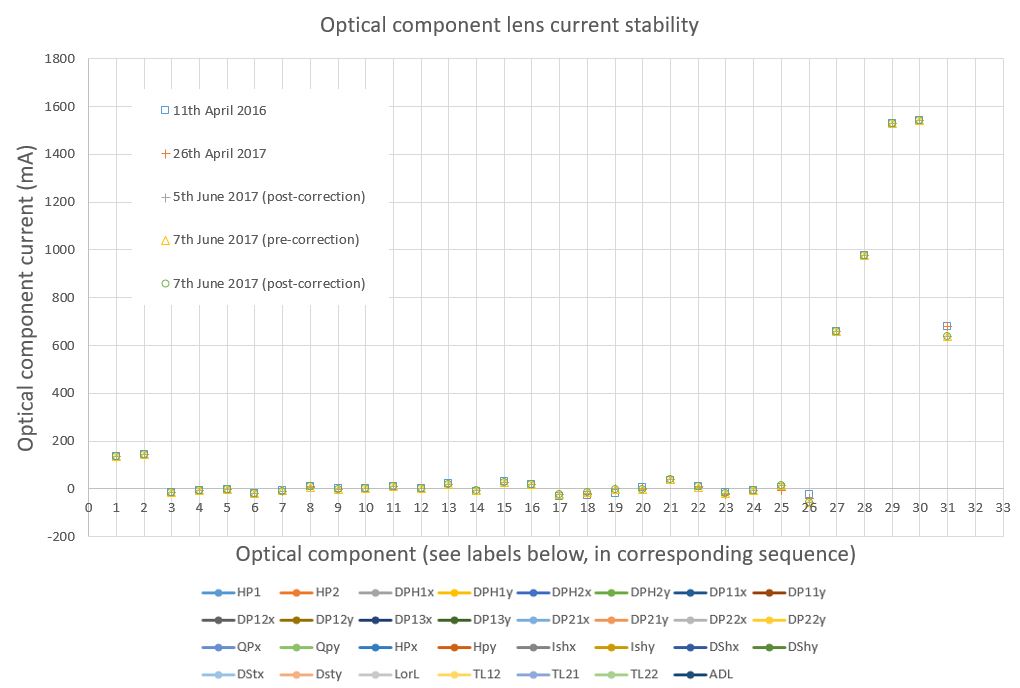

Supplement: S8 Fig — There is no discernible shift in the current drawn by any of the components, at the corrected condition. (JPG) [file pone.0186899.s014.JPG]
